# RrA, an enzyme from *Rhodospirillum rubrum*, is a prototype of a new family of short‐chain L‐asparaginases

**DOI:** 10.1002/pro.4920

**Published:** 2024-03-19

**Authors:** Di Zhang, Honorata Czapinska, Matthias Bochtler, Alexander Wlodawer, Jacek Lubkowski

**Affiliations:** ^1^ Center for Structural Biology National Cancer Institute Frederick Maryland USA; ^2^ Laboratory of Structural Biology International Institute of Molecular and Cell Biology Warsaw Poland; ^3^ Institute of Biochemistry and Biophysics Polish Academy of Sciences Warsaw Poland

**Keywords:** asparaginases, bioinformatics, biophysics, new family of enzymes, structures

## Abstract

L‐Asparaginases (ASNases) catalyze the hydrolysis of L‐Asn to L‐Asp and ammonia. Members of the ASNase family are used as drugs in the treatment of leukemia, as well as in the food industry. The protomers of bacterial ASNases typically contain 300–400 amino acids (typical class 1 ASNases). In contrast, the chain of ASNase from *Rhodospirillum rubrum*, reported here and referred to as RrA, consists of only 172 amino acid residues. RrA is homologous to the N‐terminal domain of typical bacterial class 1 ASNases and exhibits millimolar affinity for L‐Asn. In this study, we demonstrate that RrA belongs to a unique family of cytoplasmic, short‐chain ASNases (scASNases). These proteins occupy a distinct region in the sequence space, separate from the regions typically assigned to class 1 ASNases. The scASNases are present in approximately 7% of eubacterial species, spanning diverse bacterial lineages. They seem to be significantly enriched in species that encode for more than one class 1 ASNase. Here, we report biochemical, biophysical, and structural properties of RrA, a member of scASNases family. Crystal structures of the wild‐type RrA, both with and without bound L‐Asp, as well as structures of several RrA mutants, reveal topologically unique tetramers. Moreover, the active site of one protomer is complemented by two residues (Tyr21 and Asn26) from another protomer. Upon closer inspection, these findings clearly outline scASNases as a stand‐alone subfamily of ASNases that can catalyze the hydrolysis of L‐Asn to L‐Asp despite the lack of the C‐terminal domain that is present in all ASNases described structurally to date.

## 1 INTRODUCTION

Asparaginases (ASNases) are enzymes that hydrolyze L‐Asn into L‐Asp and ammonia. They are widespread across the tree of life. Many ASNases can also hydrolyze L‐Gln to L‐Glu and NH_3_, with a varying efficiency of this side reaction. During the last 60 years, different ASNases have been extensively studied and several classifications of ASNases have been proposed (da Silva et al., 2021; Loch & Jaskolski, 2021). One of the most compelling classification divides ASNases into three classes (da Silva et al., 2021). Class 1 comprises enzymes that are significantly homologous to either type I or type II ASNases from *Escherichia coli*. Division between type I and type II ASNases is based on their cellular location and on sequence similarity criteria (Lubkowski & Wlodawer, 2021). Type I ASNases are cytoplasmic enzymes that have moderate affinity for L‐Asn (in the millimolar range). Class 2 and Class 3 enzymes are not structurally related to Class 1 ASNases and, although they are capable of hydrolyzing L‐Asn to L‐Asp, that might not be their primary function.

Whereas most type I ASNases form homotetramers (Yun et al., 2007), those originating from extremophilic bacteria exist natively as homodimers (Yao et al., 2005). There is also a report of these enzymes being hexamers (Pritsa & Kyriakidis, 2001), but it was never confirmed. Type II ASNases have been described as secreted enzymes with a low‐micromolar affinity for L‐Asn. All type II ASNases characterized to date form tetramers that can be best described as pairs of intimate (tight) dimers (Swain et al., 1993), each being structurally similar to equivalent dimers of type I ASNases (Yao et al., 2005). Dissimilar class 2 and 3 ASNases have been comprehensively described in several reviews (Brannigan et al., 1995; da Silva et al., 2021; Hejazi et al., 2002; Larsen et al., 2001; Michalska et al., 2008; Moreno‐Enriquez et al., 2012; Prahl et al., 2004). Unless otherwise stated, only class 1 ASNases are discussed here.

Two type II ASNases, EcAII from *E. coli* and ErA from *Erwinia chrysanthemi* (bacterium now renamed *Dickeya dadantii*), are in clinical use for the treatment of acute lymphoblastic leukemia, lymphosarcoma, and reticulosarcoma (Avramis & Tiwari, 2006; Egler et al., 2016; Rizzari et al., 2013). When administered, these enzymes lower the plasma levels of L‐Asn, starving cancer cells that are deficient in asparagine synthetase (Aslanian & Kilberg, 2001; Prager & Bachynsky, 1968; Tabe et al., 2019). The ASNase treatment is often associated with a range of side effects. Some of those are directly linked to asparaginase‐induced accumulation of ammonia levels (Strickler et al., 2018) and are therefore inevitable. Other side effects, however, may be avoidable or at least mitigatable. Cytotoxicity related to residual glutaminase activity of ASNases (Parmentier et al., 2015) may be minimized for asparagine synthetase‐negative leukemias by using enzymes with minimal glutaminolytic activity (Chan et al., 2019). Induced hypersensitivity may be addressed by a change of the enzyme during treatment to evade the immune response (Rizzari et al., 2013).

The quest for new ASNases with potentially favorable pharmacological properties has motivated the characterization of many of these enzymes, including an unusual asparaginase from *Rhodospirillum rubrum*. This enzyme, referred to here as RrA, is about half the size of a typical class 1 ASNase and its amino acid sequence is similar to the N‐terminal domain of class 1 ASNases (Pokrovskaya et al., 2012). The affinity of RrA for L‐Asn was initially reported to be in the mid‐micromolar range (Pokrovskaya et al., 2012; Pokrovskaya et al., 2015), later corrected to low millimolar (Dobryakova et al., 2022). Therefore, unmodified RrA is unsuitable for anticancer therapy. Interestingly, however, modest antitumor activity of a variant of RrA was detected in a mouse xenograft model and attributed to the telomerase inhibition utilizing an unknown mechanism (Zhdanov et al., 2017). So far, only unsuccessful attempts to improve the pharmacological properties of RrA have been reported (Dobryakova et al., 2022).

Here, we demonstrate that RrA is not a unique anomaly among L‐Asn‐hydrolyzing enzymes, but represents a previously unrecognized family of short cytoplasmic ASNases (scASNases). To ensure proper distinction, the previously described ASNases will be referred to as ‘typical class 1 ASNases’. In this work, we determined crystal structures of wild‐type RrA and its several variants and explored some of their basic physicochemical and biochemical properties. We confirmed experimentally the previously modeled tetrameric architecture of the enzyme (Dobryakova et al., 2022). We have also demonstrated that RrA exists as a tetramer in solution even at picomolar concentrations. Crystal structures of this scASNase show that its complete active site is formed by two protomers within a tetramer.

## 2 RESULTS

### 2.1 A family of short ASNases

**FIGURE 1:**
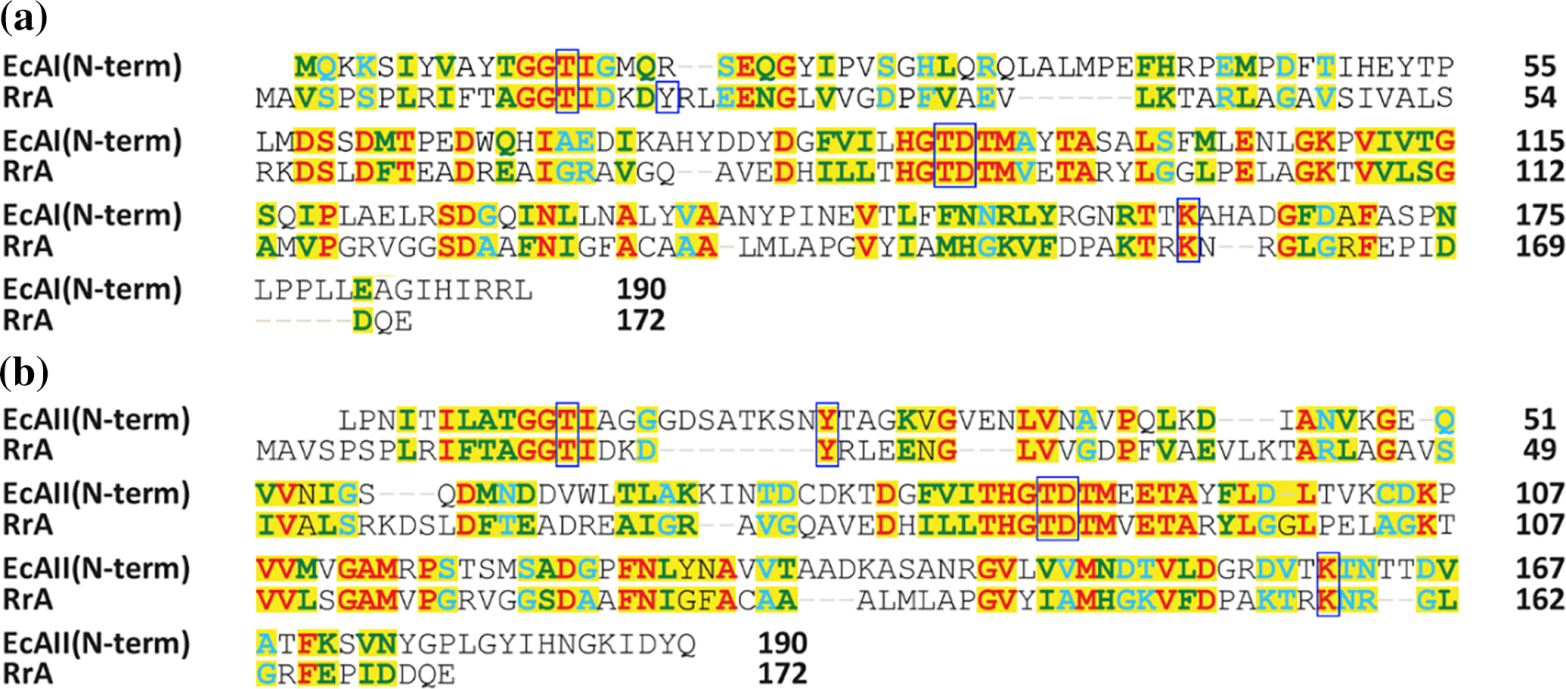
Amino acid sequence alignment. Alignment of the amino acid sequences of RrA and the N‐terminal domain of either (a) EcAI or (b) EcAII. The sequences were aligned using the program Clustal Omega (Sievers & Higgins, 2021). Homologous residues are highlighted with a yellow background. Identical residues are printed in red, those highly similar in green, and weakly similar residues are printed in cyan. Residues known to be crucial for the catalytic activity of EcAI and EcAII are enclosed in blue frames.

The amino acid sequence of RrA aligns modestly well with the sequences of class I ASNases (Figure 1). The reported low affinity for L‐Asn (Dobryakova et al., 2022), and the unusually short protein chain, led us to consider a possibility that the amino acid sequence of the *R. rubrum* protein might have resulted from a truncation causing a nonsense mutation or a frameshift. Such a mutation could have occurred naturally in *R. rubrum* or might have been erroneously inferred due to a sequence or assembly error. However, re‐analysis of the raw sequence data supports the deposited nucleotide sequence (Munk et al., 2011). Moreover, upon inspection, it became evident that the downstream genomic neighbor of RrA is positioned too close for a C‐terminal fragment to fit into the intervening space in any frame. TBLASTN (Gertz et al., 2006) searches using the nucleotide sequences of the C‐terminal region of *E. coli* type I (*asnA*) and type II (*asnB*) enzymes as the queries further demonstrated that the rest of the genome also does not contain an open reading frame for a stand‐alone homologue of the C‐terminal fragment of scASNases, ruling out RrA heterodimers that might resemble typical asparaginases. Therefore, RrA could either be a ‘one‐off’ protein resulting from a truncation of a class 1 ASNase in *R. rubrum* only, or it could be a representative of a larger family of scASNases, previously uncharacterized.

**FIGURE 2:**
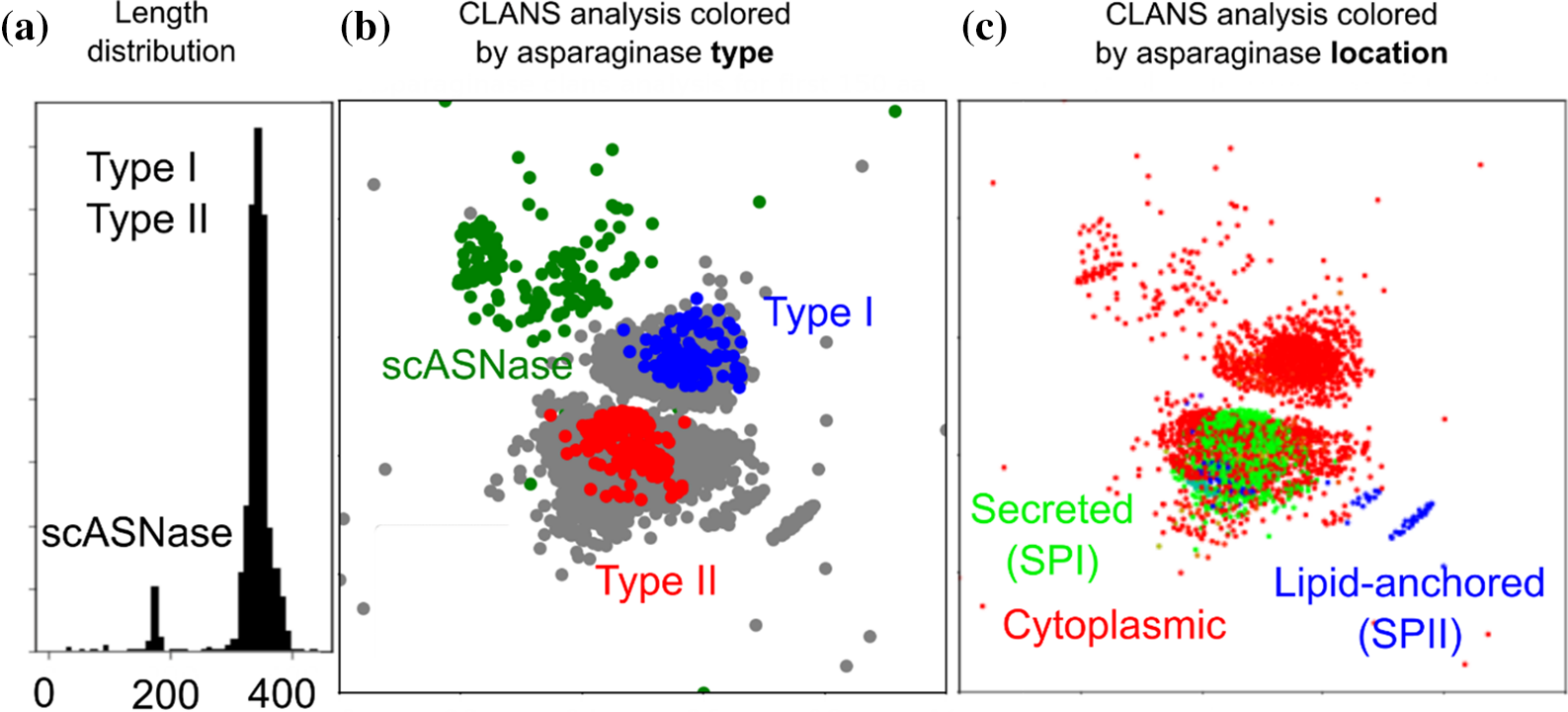
Asparaginase families. (a) ASNase length distribution. The amino acid sequences of EcAI, EcAII, and RrA were used for TBLASTN searches (*E*‐value 0.01) for asparaginases in microbial genomes. Altogether, 3860 GenBank files representing 1554 distinct species from the EMBL collection were searched. Based on the aligned regions, open reading frames (ORFs) were determined, and the length distribution of these ORFs (not of the aligned regions) was plotted. The bimodal length distribution demonstrates that scASNases form a separate group that is clearly distinct from the group of long asparaginases. (b) CLANS (Frickey & Lupas, 2004) analysis of ASNases, colored according to type. To exclude any influence of sequence length on the clustering, only the first 150 amino acid sequences were considered. Every dot represents one asparaginase protein. Long asparaginases are colored gray, if they were previously either unannotated, or only generically annotated as ASNases. The data show that the scASNases form a phylogenetically separate group from the class I ASNases. (c) CLANS (Frickey & Lupas, 2004) analysis as in (b), but colored according to predicted cellular localization, according to SignalP6.0 (Teufel et al., 2022). ASNases without a predicted signal peptide are presumed to be cytoplasmic. Enzymes harboring an SPase I signal peptide are expected to be secreted to the extracellular milieu, or—in case of gram negative bacteria—also to the periplasm (Natale et al., 2008; Teufel et al., 2022). Those with an SPase II signal peptide are expected to be lipid anchored (Natale et al., 2008; Teufel et al., 2022). The data show that scASNases, like type I ASNases, are cytoplasmic. The data also indicate that type II asparaginases have mixed localization, and are not all universally secreted, as the literature suggests.

In order to investigate the content of class 1 bacterial ASNases, we queried 3860 complete genomes representing 1554 different bacterial species (EMBL accessions, species names, and asparaginase counts are provided in the Supplementary Excel S3 [Genome accessions]). We used type I and type II ASNases, as well as the sequence of RrA, as search sequences. Based on the TBLASTN hits (with an *E*‐value cut‐off of 0.01), we extracted full length open reading frames and translated them to the protein sequence. The length distribution of the predicted proteins was bimodal. Most of the ASNases consisted of 300–400 amino acids, representing typical class 1 enzymes. However, there was also a distinct second group of putative shorter ASNases (scASNases) with a length of 160–180 amino acids, found in approximately 7% of the investigated bacterial species (Figure 2a). These enzymes were identified in both gram‐negative and gram‐positive bacteria. In our ASNase dataset, we found instances of scASNases in the phyla *Acidobacteria*, *Bacteroidetes*, *Chlorobi*, *Deinococcus‐Thermus*, *Firmicutes*, *Gemmatimonadetes*, *Proteobacteria*, and *Spirochaetes*. Despite their overall scarcity, the widespread distribution of scASNases is reminiscent of the scattered distribution of typical class 1 ASNases (Zielezinski et al., 2022) and suggests frequent horizontal gene transfer events.

### 2.2 Short ASNases form a distinct phylogenetic group

To clarify the relationship between scASNases and the class 1 enzymes, we conducted a CLuster ANalysis of Sequences (CLANS) analysis (Frickey & Lupas, 2004). CLANS analysis creates a graph representation of pairwise sequence similarities that are expressed as proximity between dots representing one sequence each. To avoid cluster formation according to sequence length, only the N‐terminal 150 amino acids were used for sequence comparisons. The analysis unveiled three main groups of ASNases. Two clusters encompassed ASNases previously annotated as type I or type II, and the third cluster consisted of scASNases. Since the clustering was based solely on the region common to all three groups, this analysis demonstrated that scASNases represent a phylogenetically distinct group that has not arisen through numerous independent protein truncation events from the longer class 1 enzymes. Instead, scASNases constitute a separate, early‐diverging group, more closely related by the sequence comparison to type I than type II ASNases (Figure 2b).

To generate hypotheses about potential cellular roles of scASNases, we searched for signal peptides using the SignalP server (Teufel et al., 2022) to predict cellular localization. Detection of signal peptidase I (SPI) and signal peptidase II (SPII) leader peptides predicts secretion and lipid anchoring, respectively, the absence of a leader peptide indicates cytoplasmic localization (Natale et al., 2008; Teufel et al., 2022). The analysis suggested that scASNases are almost universally cytoplasmic, similar to type I ASNases. Interestingly, the cluster of type II ASNases was predicted to have mixed cellular localization, with some proteins predicted to be cytoplasmic, and a few to be lipid anchored, in contrast to the widely accepted consensus identifying the enzymes from this group as secreted (Figure 2c). To validate the cytoplasmic localization of some ASNases that by sequence would be classified as type II, we scanned the eSORTdb database of proteins with experimentally validated localization (Lau et al., 2021) for asparaginases. Six class 1 ASNases were found, one cytoplasmic type I enzyme, and five type II enzymes. Among the latter ones, three were listed as secreted to the periplasm, as the literature would suggest, whereas two, from gram‐positive *Bacillus lichiformis* (gi|231,572, SWISSPROT P30363) and gram‐negative *Wolinella succinogenes* (gi|37,154,620, SWISSPROT P50286), were listed as cytoplasmic.

**FIGURE 3:**
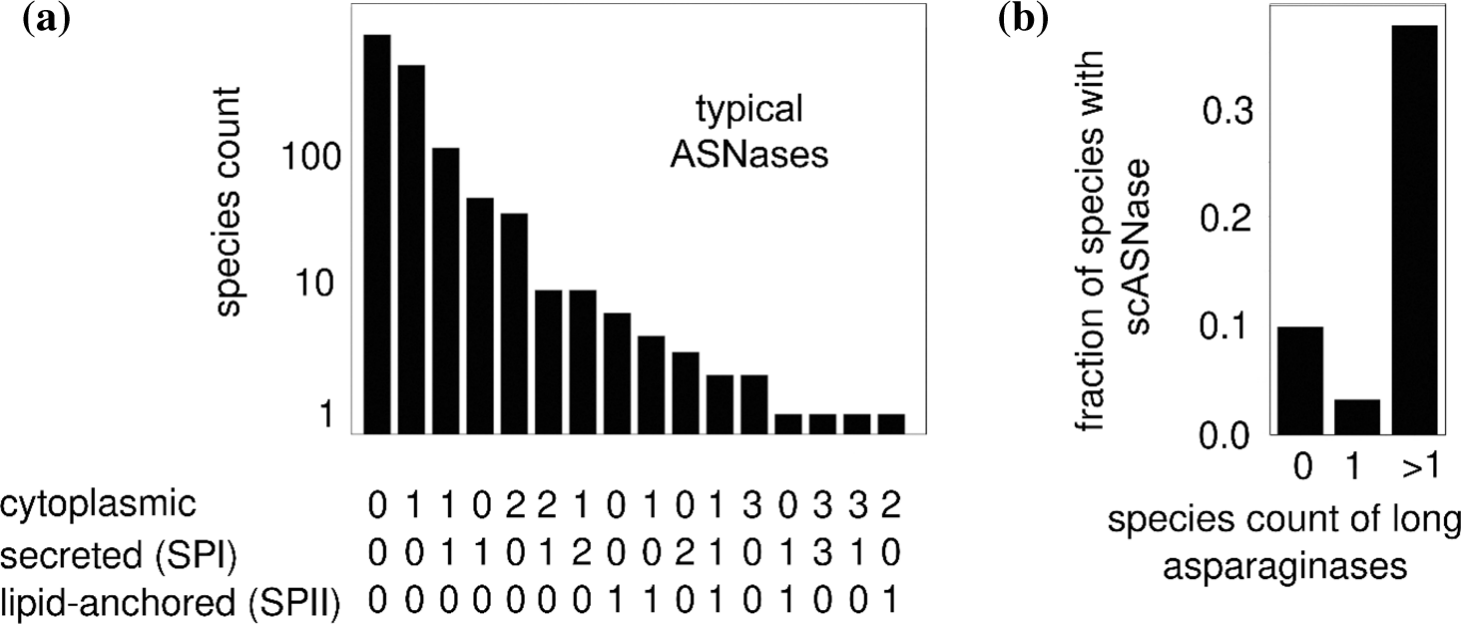
Asparaginase co‐occurrence. (a) Co‐occurrence of typical (long chain) ASNases (lcASNases). For every bacterial species, a tally was taken for cytoplasmic, secreted (SPI) and lipid‐anchored (SPII) ASNases. The number of bacterial species with a given repertoire of lcASNases was then counted (note the logarithmic scale of the ordinate). The data indicate that the most common scenario (per organism) is “no ASNase at all”, followed by “one cytoplasmic lcASNase”, and “one cytoplasmic and one secreted lcASNase each”. (b) Predictive value of lcASNase content for scASNase content. Bacterial species were grouped according to the number of lcASNases in their genomes (0, 1, or >1). In each group, the fraction of species was counted that contained at least one scASNase. The data show that almost 40% of bacterial species with more than one lcASNase also contain a scASNase, whereas this proportion is much lower for bacterial species with either no or only a single lcASNase. Our analysis suggests that bacterial species that depend on asparaginase activity accumulate both lc and scASNases.

The co‐occurrence of scASNases and class 1 ASNases may hold clues about the function of scASNases. As a baseline, we first investigated the co‐occurrence of typical (i.e. long) class 1 ASNases. The largest fraction of bacteria lacked class 1 ASNase. In other bacteria, the most common scenarios were (i) a single cytosolic ASNase, followed by (ii) one cytosolic and one secreted enzyme, and (iii) a single secreted ASNase, aligning with recent observations from an asparaginase survey (Zielezinski et al., 2022). Different combinations of ASNases could be present within a single bacterium, including predicted cytoplasmic, secreted, and lipid‐anchored ASNases. Some genomes encoded multiple cytoplasmic ASNases alongside several secreted enzymes (Figure 3a). We then examined whether the repertoire of class 1 ASNases predicted the presence or absence of scASNases. Bacteria carrying just one class 1 ASNase had the lowest frequency of genes coding for short‐chain enzymes. The occurrence of scASNase genes was slightly higher in bacteria without any class 1 ASNase, and much higher, almost 40%, for bacteria encoding more than one class 1 ASNase (Figure 3b).

**FIGURE 4:**
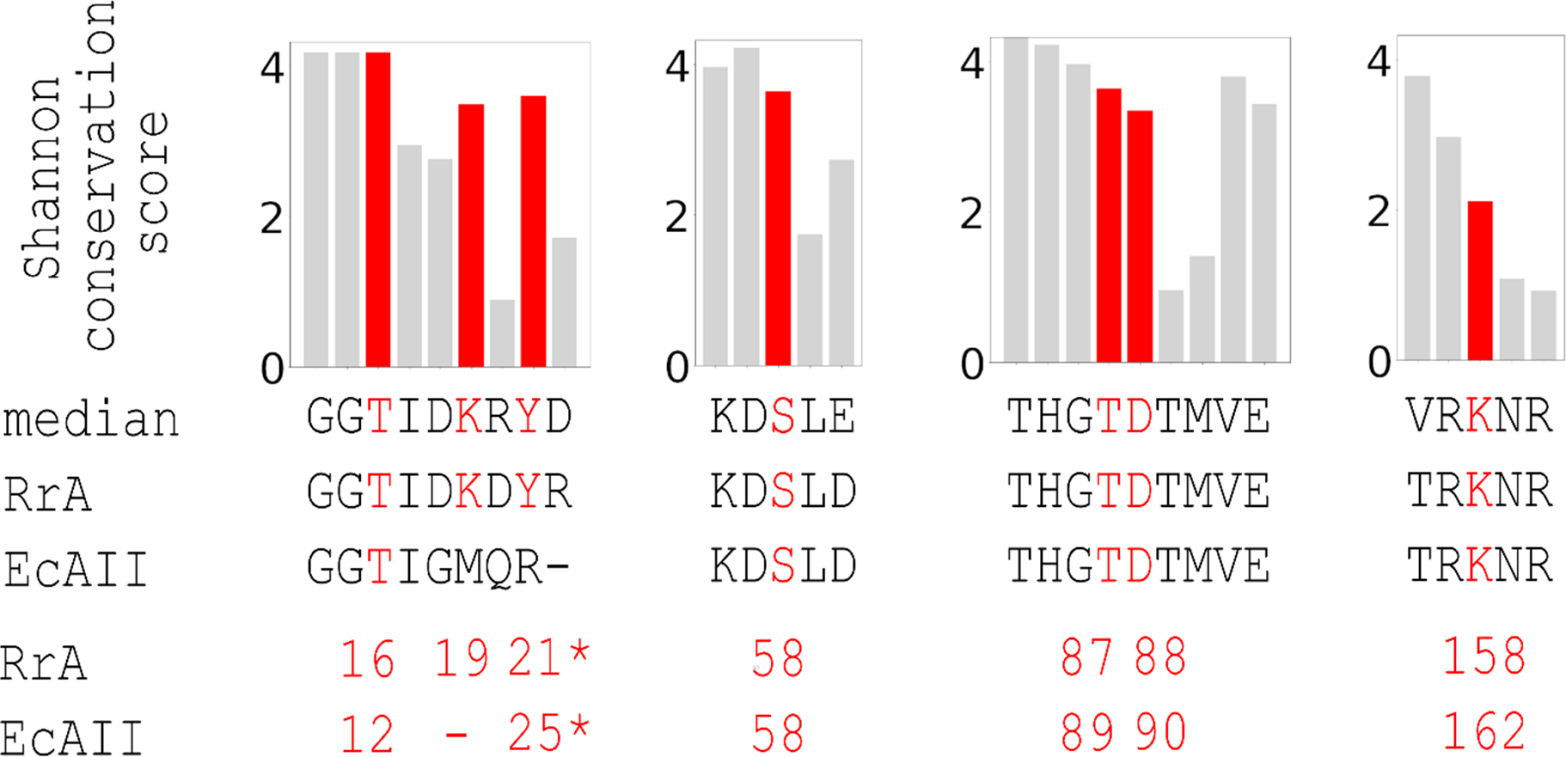
Conservation of key asparaginase residues. Representative amino acid sequences of scASNases were selected using CD‐HIT (clustering threshold 0.9) (Li & Godzik, 2006), aligned, and analyzed for sequence conservation using the Shannon score, defined as the difference between the maximally possible and actual sequence entropy (in bits). A Shannon score of log_2_20 ~ 4.3 indicates perfect conservation, a Shannon score of 0 indicates a uniform representation of amino acids in a given position of the sequence. Residues discussed in the text are indicated in red. “Median” indicates the consensus sequence for the scASNase family, defined as the Levenshtein median string for all sequences in the alignment. The “*” symbols indicate that Tyr21 of RrA and Tyr25 of EcAII are structurally equivalent, but anchored in non‐equivalent positions of the amino acid backbone.

To investigate the conservation of residues forming the active sites, we selected 81 representative scASNases using CD‐HIT (Li & Godzik, 2006) (with a 0.9 grouping threshold) and, after aligning them, calculated Shannon conservation scores. According to this analysis, the putative N‐terminal active site threonine (Thr16 in RrA), predicted to be responsible for the initial acylation reaction in class 1 ASNases (see below), is strictly conserved in scASNases (Figure 4a). The more C‐terminal active site threonine (Thr87 in RrA), important during the deacylation reaction in class 1 ASNases, is highly, although not perfectly, conserved. The same applies to the immediately adjacent aspartate residue (Asp88) (Figure 4b). Exceptional cases from *Zunongwandia profunda*, *Gramella forsetii*, and *Celeribacter indicus* had non‐functional replacements for the TD motif (IF, IF and TG, respectively). Collectively, these data suggest that at least some asparaginase active site residues are subject to evolutionary selection. Thus, we anticipate that scASNases constitute a group of catalytically active enzymes.

### 2.3 Quaternary structure of RrA in solution

The oligomerization of RrA in solution was assessed using two methods: dynamic light scattering (DLS) and mass photometry (MP). The DLS experiments were conducted at pH 7.0 and relatively high RrA concentrations, varying between 0.28 and 1.1 mM. The results of these experiments are presented in Table 1. An analysis using the manufacturer's provided software suggests that all studied RrA variants exist in solution as homotetramers. However, it is important to note that the DLS analysis does not account for the molecule shape, resulting in approximate values for the hydrodynamic radius (Rh) of individual species (monomer, dimer, or tetramer). Thus, this approach limits the ability to classify species with similar Rh values into distinct clusters.

**TABLE 1:** Studies of RrA variants at different concentrations using the dynamic light scattering.

RrA(wt)				RrA(Y21F)			
Concentration (mM)	Rh (nm)^a^	Pd (%)^b^	MW (kDa)^c^	Concentration (mM)	Rh (nm)^a^	Pd (%)^b^	MW (kDa)^c^
1.1	3.6	9.1	67.6	0.55	3.7	5.0	71.2
0.22	3.7	5.5	71.6	0.28	3.6	5.1	69.4

RrA(Y21A)				RrA(K158M)			
Concentration (mM)	Rh (nm)^a^	Pd (%)^b^	MW (kDa)^c^	Concentration (mM)	Rh (nm)^a^	Pd (%)^b^	MW (kDa)^c^
1.1	3.3	14.1	55.1	1.1	3.8	16.7	78.4
0.28	3.3	21.5	53.6	0.28	3.8	9.	75.5

RrA(K19A)				RrA(K19E)			
Concentration (mM)	Rh (nm)^a^	Pd (%)^b^	MW (kDa)^c^	Concentration (mM)	Rh (nm)^a^	Pd (%)^b^	MW (kDa)^c^
1.1	3.6	6.1	66.6	1.1	3.7	11.2	71.3
0.22	3.8	8.6	75.3	0.22	3.7	7.7	73.9

RrA(K19Q)			
Concentration (mM)	Rh (nm)^a^	Pd (%)^b^	MW (kDa)^c^
1.1	3.5	8.1	64.2
0.22	3.9	19.1	80.1

*Note*: Modeling proteins as globular is the best available approximation for the software used here.

^a^ Hydrodynamic radius.

^b^ Polydispersity, calculated as the ratio between standard deviation of a peak (represented as a histogram of results from multiple scans) and the mean value of this peak, expressed in percentage (Pd < 15% suggests monodispersity of a sample).

^c^ This is estimated MW, theoretical MWs of oligomers are: 18.2 kDa (monomer), 36.3 kDa (dimer), and 72.6 kDa (tetramer).

MP measurements for RrA(wt) were conducted at low enzyme concentrations, here at 25 nM and 65 nM, using the standard buffer (50 mM HEPES, 200 mM NaCl, pH 7) at 20°C. Under the conditions of the assay, we observed that RrA is primarily tetrameric (Figure S1). However, a minor peak at lower mass observed in the histogram for the 25 nM sample (Figure S1C) suggests the presence of a small dimeric fraction.

### 2.4 Crystal structure of RrA

**FIGURE 5:**
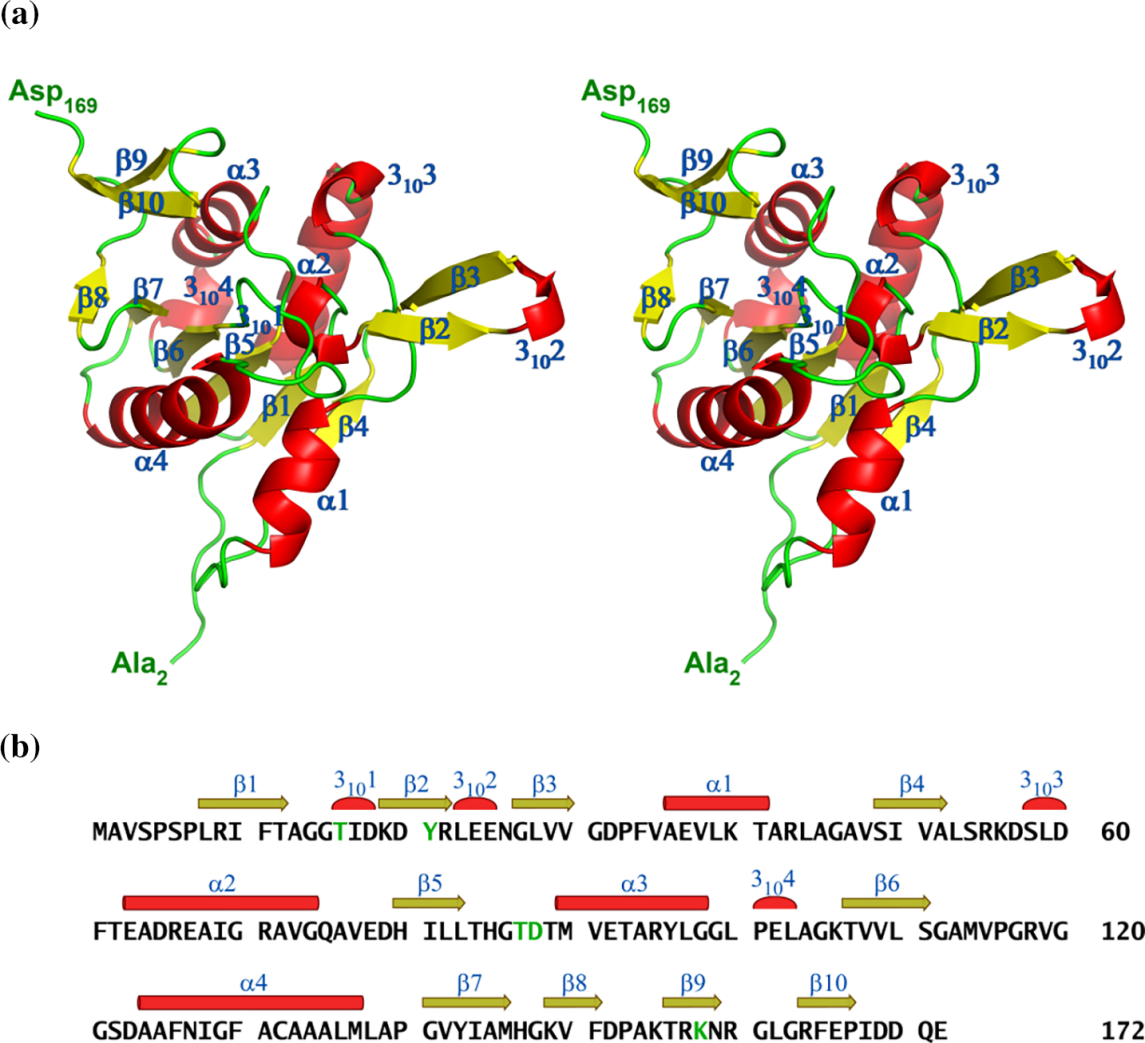
Structure of RrA. (a) The cartoon representation of the RrA protomer depicted in stereo view. The β‐strands, labeled β1 through β10, are shown in yellow, while α‐ and 3_10_‐helices, labeled α1 through α4 and 3_10_1 through 3_10_4, respectively, are displayed in red. The secondary structure elements were assigned using the DSSP program (Kabsch & Sander, 1983). Both termini are indicated. (b) The primary structure of RrA is illustrated with secondary structure elements indicated above the sequence and labeled. Five residues identified as critical for catalysis are highlighted in green. In RrA, these residues are Thr16, Tyr21, Thr87, Asp88, and Lys158. Panel A was generated using the program Pymol 2.5.2 (Schrödinger, LLC).

**FIGURE 6:**
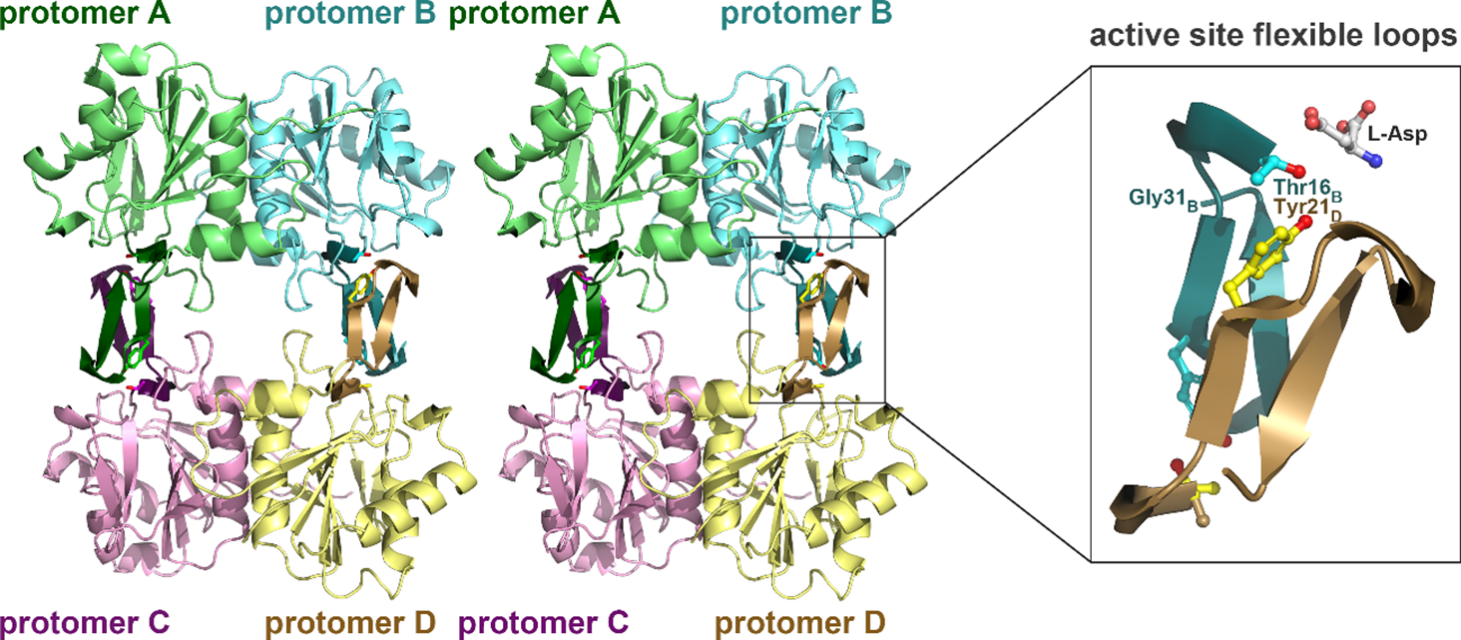
Tetramer of RrA. The stereo view of the RrA homotetramer. Individual protomers are uniquely colored. The tetramer can be envisioned as the dimer of tight dimers (here green/cyan and magenta/yellow). Dimers interact with each other via the active side hairpins, composed of strands β2 and β3 that form the active site flexible loop (ASFL). These flexible loops are colored in darker shades than their contributing protomers. In contrast to type I ASNases, where the active sites are formed by residues from two protomers within a tight dimer, in RrA two protomers from different tight dimers contribute to any of the active sites. Interaction of hairpins from such two protomers is shown in the box at the right side of the figure. In RrA, ASFL extends from Thr16 to Gly31 and contributes two residues, Thr16 and Tyr21, that are important for the enzymatic activity. Side chains of both residues, together with L‐Asp, the product of catalytic reaction, are shown in stick representation and labeled.

Crystals of RrA(wt) were grown in the space group *P*2_1_2_1_2, with two protomers in an asymmetric unit (a.u.). Isomorphous crystals grew both in the absence and presence of L‐Asp. A protomer of RrA consists of 172 amino acid residues with a fold very similar to the one of the N‐terminal domain of a typical class 1 ASNase (Lubkowski & Wlodawer, 2021) (Figure 5). In the structure of RrA(wt) in complex with L‐Asp, protomer A was traced in the electron density from Met1 through Asp169, whereas protomer B was traced from Ser6 through Ile168. Superposition of protomer B onto A resulted in a root‐mean‐square deviation (rmsd) of 0.48 Å for 162 equivalent Cα atoms. In the structure of the ligand‐free enzyme, residues 58–60 of protomer A were not traced due to the lack of interpretable electron density. The two protomers form a tight dimer (designated AB in the structure) with an interface area of approximately 1100 Å^2^. The tetramer (Figure 6) was created by 2‐fold crystal symmetry, with intermolecular contacts observed only between protomers A‐A' and B‐B′, yielding an interface area of around 625 Å^2^ for each pair.

The overall architecture of the RrA molecule is that of a 3‐layer (αβα) sandwich with the α/β topology, often referred to as the Rossmann‐like motif (Rossmann et al., 1974). Six β‐strands, located at the center of this motif, form a mixed β‐sheet (Hutchinson & Thornton, 1996) with strand connectivity [−1x, +2x, +1x, +1x, +1x] (Richardson, 1977), formed by sequentially numbered strands 4‐1‐5‐6‐7‐8 (Figure 5). The crossover between β6 and β7, which contains helix α4, is left‐handed, a feature seldom found in proteins but described before for typical class 1 ASNases (Miller et al., 1993). The pair of strands β2 and β3 forms a hairpin with a 3_10_‐helical motif at its apex. There are two α‐helices on either side of the central β‐sheet: α1 and α4, or α2 and α3. Additionally, two 3_10_‐helical turns intersperse the aforementioned secondary structure elements. Both termini have a coil structure and are likely disordered in the native state.

### 2.5 Structural comparison of RrA(wt) with typical class 1 ASNases

**FIGURE 7:**
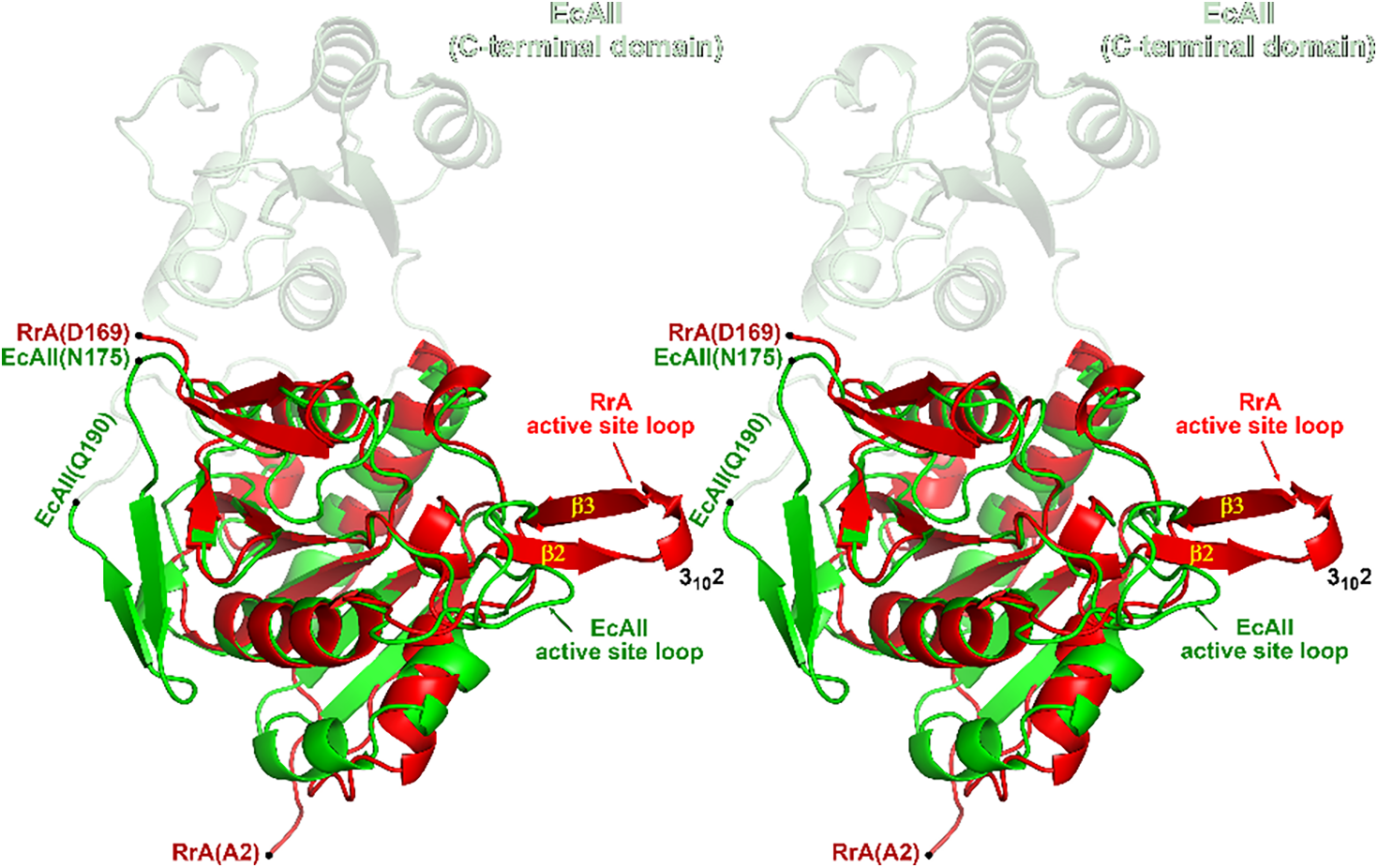
Superposition of RrA and the N‐terminal domain of EcAII. RrA (protomer A, shown in red, in structure of RrA(wt), PDB entry 8uou) and the N‐terminal domain of EcAII (protomer A in PDB entry 3eca, green) are portrayed in a cartoon representation. In the case of EcAII, the N‐terminal domain (residues 1–190) is shown in solid green, while the smaller C‐terminal domain (residues 191–326), included for reference, is depicted in semi‐transparent form. Relevant sites/motifs/secondary structure elements are labeled. The figure was prepared using the program Pymol 2.5.2 (Schrödinger, LLC).

Superposition of the protomer A of RrA(wt) with residues 1–175 of EcAII (PDB ID 3eca, chain A) results in the rmsd of 1.7 Å for 153 equivalent Cα pairs, despite a sequence identity of only 26.1% (Figure 1b). An evident structural difference arises in the central β‐sheet (Figure 7). In EcAII, this motif forms an eight‐stranded mixed sheet. The N‐terminal domain of EcAII is nearly 20 residues longer than the chain of RrA, and the last two β‐strands within the central sheet of EcAII are formed by the additional residues, Asn175‐Gln190 (Figures 1b and 7). Two other segments that exhibit distinct topologies in both enzymes are the hairpins β2‐3_10_2‐β3 and β9‐3_10_5‐β10 in RrA, which correspond to coil‐like motifs in EcAII. It is worth noting that in typical class 1 ASNases the former segment represents the active site flexible loop (ASFL) (Lubkowski & Wlodawer, 2021). This fragment contains two important activity‐related residues, a threonine (Thr16 in RrA) and a tyrosine (Tyr21 in RrA). The second region provides the catalytic lysine residue (Lys158 in RrA) (Figures 1a,b and 5b). Earlier studies demonstrated that in typical ASNases, conformations of the side chains contributed by these residues depend on the occupancy of the active site (Lubkowski & Wlodawer, 2021; Strzelczyk et al., 2020).

Although the topologies of the RrA protomer and the EcAII N‐terminal domain are similar, the ASFL fragment extending from Lys19 through Gly31 of RrA points in an entirely different direction compared to its counterpart in EcAII (Gly15 through Lys29), with the distance between residues at the tips of the loops exceeding 20 Å. The ASFL loop is highly flexible in type II ASNases, and in the absence of a ligand in the active site it is typically disordered. However, the corresponding loop in RrA remains fully ordered regardless of the active site occupancy, and its temperature factors are comparable to those of the rest of the protein chain. Active site loops from different protomers create interfaces between two tight dimers, thereby stabilizing the tetramer. The involvement of active site loops in tetramer formation may be directly linked to the rigidity of these motifs. While the role of RrA tetramerization is not entirely clear, its homotetramers are observable by MP at concentrations as low as 25 nM.

### 2.6 Structures of RrA mutants

Several single‐site mutants of RrA were expressed, purified, and crystallized. Residues Lys19, Tyr21, and Lys158 were chosen to be mutated, because they were expected of playing accessory roles in catalysis. According to the crystal structure of the wild‐type protein, Lys19 and Tyr21 might be involved in the proton shuttle from the N‐terminal active site threonine. Mutation of Lys158 was anticipated to impede the completion of the catalytic event by preventing the second step of the reaction that would result in hydrolysis of the covalent acyl‐enzyme intermediate (Lubkowski et al., 2020). Analysis of sequence conservation within the scASNase family indicated that these residues are either mostly (Lys19, Tyr21), or only moderately (Lys158) conserved (Figure 4).

Crystallization of the variants in the absence or presence of L‐Asp yielded three different crystal forms. In most cases, we obtained crystals in orthorhombic space group *P*2_1_2_1_2, with a tight dimer in the a. u. In this case a tetramer was created by two‐fold crystallographic symmetry. In some cases, orthorhombic crystals with a doubled *c* axis were obtained, belonging to space group *P*2_1_2_1_2_1_. The a. u. for these crystals contained a complete tetramer. Finally, the Y21F variant grown in the absence of L‐Asp crystallized in space group *C*2 with two complete tetramers in the a. u. (Table 2). Protomer structures were largely similar in all cases, with the main differences occurring in loops around residues 20 (ASFL) and 60.

**TABLE 2:** Abbreviated statistics of data collection and structure refinement.

Structure RrA	Mut	L‐asp	Space group	Asymmetric unit	Resolution (Å)	*R*_cryst_	*R*_free_	PDB
wt	−	+	*P*2_1_2_1_2	(A, B)	1.35	0.125 (0.251)	0.170 (0.301)	8uou
	−	−			1.65	0.188 (0.251)	0.222 (0.256)	8uoo
K158	M	−			1.20	0.140 (0.391)	0.164 (0.430)	8upc
K19	E	−			1.45	0.123 (0.210)	0.166 (0.289)	8uor
	Q	−			1.47	0.128 (0.193)	0.169 (0.253)	8up9
	A	−			1.70	0.200 (0.282)	0.233 (0.313)	8uow
	A	+	*P*2_1_2_1_2_1_	[(A, B), (C, D)]	1.70	0.182 (0.230)	0.205 (0.280)	8up6
Y21	A	−			1.59	0.187 (0.221)	0.221 (0.273)	8up7
	F	+			1.90	0.179 (0.193)	0.225 (0.270)	8up8
	F	(+)	*C*2	[(A, B), (C, D)] [(E, F), (G, H)]	1.76	0.165 (0.219)	0.216 (0.209)	8up3

*Note*: (+) indicates that L‐Asp was not added to the crystallization mix, but density for L‐Asp was nonetheless found in some ASNase subunits in the asymmetric unit. Molecules within parentheses denote tight dimers, and within square brackets denote tetramers.

#### 2.6.1 K19 variants

Crystals of RrA(K19Q) were grown both in the absence and presence of L‐Asp in the crystallization buffer. However, aspartate could not be modeled in either structure, although some density found in the active sites appeared to represent molecules other than water. Gln19 interacts indirectly with the OH of Tyr21 of an adjacent protomer through a water molecule. There were no disordered regions in the main chain of any protomer, aside from some N‐ and C‐terminal residues. Crystals of RrA(K19E) were isomorphous with those of RrA(K19Q) and were obtained only in the absence of L‐Asp. Crystals of RrA(K19A) obtained in the absence of L‐Asp were also isomorphous with those of RrA(K19Q). The region 58–62 of the main chain of protomer A of RrA(K19A) is disordered. However, the main chain adjacent to the mutation site is well ordered in both protomers, and the area occupied by the side chain of Lys19 in the native protein appears occupied only by water molecules.

#### 2.6.2 Y21 variants

RrA(Y21A) without added L‐Asp was disordered in the region of residues 22–26 and the active site contained a mostly disordered HEPES molecule contributed by the buffer. Solvent molecules occupied the space where the Tyr side chain is present in RrA(wt). RrA(Y21F), also grown without added L‐Asp, nonetheless contained L‐Asp in the active sites of some protomers, presumably co‐purified from the cell lysate. Other protomers contained water molecules, ethylene glycol, and/or chloride ions, all contributed by the buffer. In all protomers without a bound L‐Asp we observed a putative H‐bond between Thr16 and OD1 of Asn26, which belongs to the same chain as Phe21. The distance between Thr16(OG1) and Lys19(NZ) was longer than in RrA(wt), exceeding 4 Å in all eight molecules. When crystallization was done in the presence of L‐Asp, the ligand was present in three out of four protomers and the main chain became fully ordered. As expected, the phenyl group of Phe21 occupied almost exactly the same position as Tyr21 in RrA(wt), leaving no room for any water molecule(s). L‐Asp was bound predominantly by one chain, except that a hydrogen bond was made between its amino nitrogen and the OD2 oxygen of Asn26 of the adjacent protomer.

#### 2.6.3 K168 variant

RrA(K168M) was crystallized in the presence of L‐Asp. However, the density present in the active site could be modeled only by several water molecules. The methionine side chains in the mutated site were modeled with two orientations of unequal occupancy. A water molecule forms a bridge between Lys19 and Thr16 of one protomer, as well as with Tyr21 of another protomer.

### 2.7 Comparison of the active sites of RrA and EcAII

**FIGURE 8:**
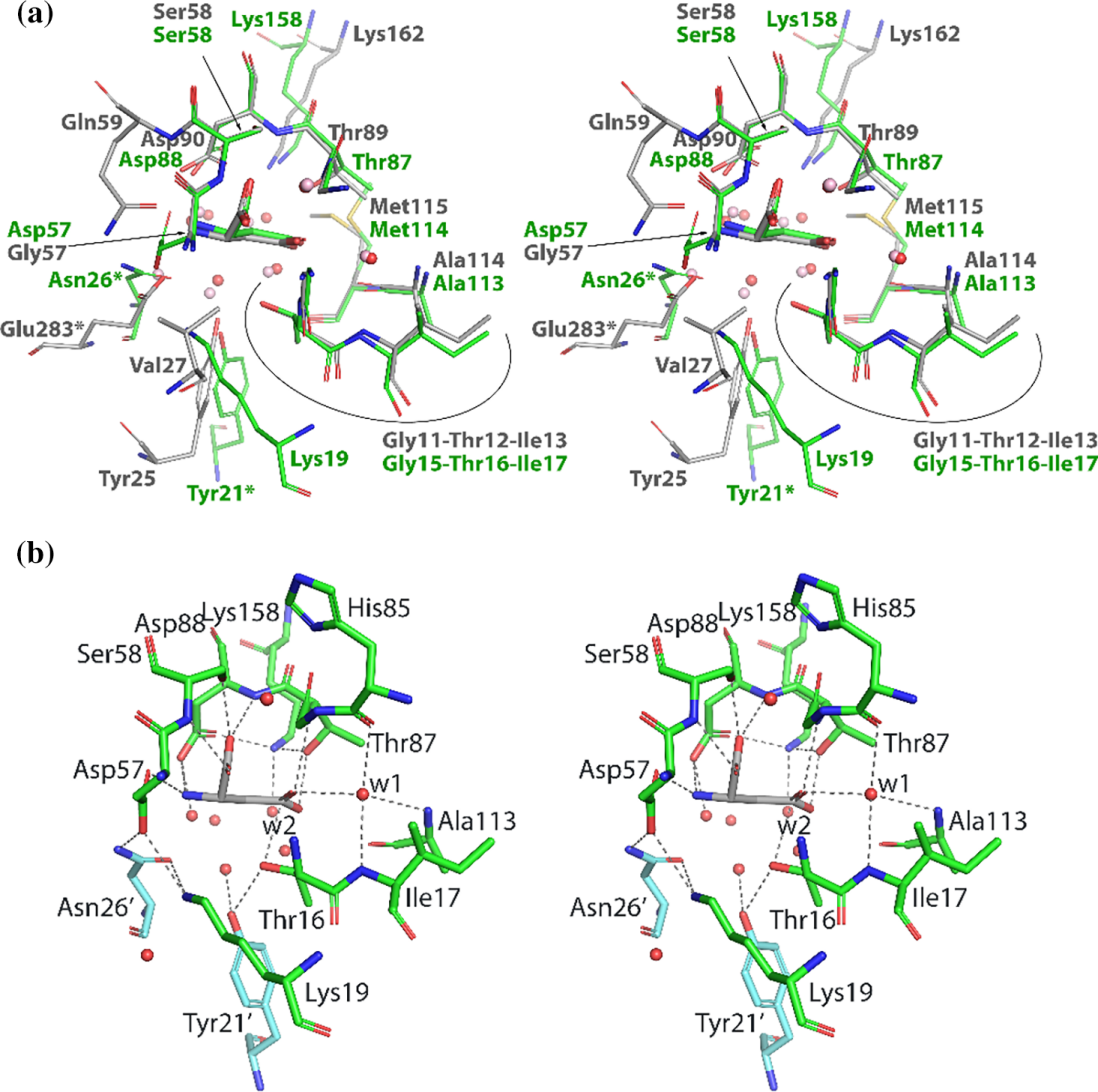
The active site of ASNases. (a) The superposition of the coordinates of L‐Asp complexes of RrA (green) and EcAII (gray). In both enzymes, the bulk of the active sites is formed by one protomer. Residues contributed by the second protomer are numbered with an asterisk. Water molecules are depicted as pink (EcAII) or red (RrA) spheres. (b) Stabilizing interactions within the active site of RrA occupied by L‐Asp (dashed lines). Water molecules that were previously found as critical for the enzymatic performance of type I ASNases and are also conserved in RrA are labeled w1 (component of the oxyanion hole) and w2 (second nucleophile) (Lubkowski et al., 2020).

The structure of RrA(wt) with L‐Asp bound in the active site (Figure 8) was used to provide a detailed description of enzyme‐substrate interactions. As shown previously (Röhm & Van Etten, 1986), L‐Asp with protonated β‐carboxylate is a substrate of ASNases. In that case fully reversible catalytic reaction is equivalent to the exchange of an oxygen atom between β‐carboxylate and a water molecule sequestered from bulk solvent. Under physiological conditions, however, L‐Asp should be considered the product of hydrolysis by ASNases. Despite the overall low sequence identity between RrA and typical class 1 ASNases, here represented by EcAII (Figure 1b), the environment of L‐Asp bound to the active site (Figure 8a) is very similar for both enzymes. As previously mentioned, Thr16 (presumed to be the primary nucleophile in RrA) remains well ordered even in the absence of a substrate. In the complex with L‐Asp, the hydroxyl oxygen of Thr16 is located approximately 2.8 Å from the γ‐carbon atom of the β‐carboxylate of the substrate (Figure 8b). The two oxygen atoms of this carboxylate are within hydrogen‐bonding distances of OG1 and the main chain N of Thr87. Oxygen atoms from the α‐carboxylate group of L‐Asp are within hydrogen‐bonding distance to the main chain N of Asp88 and OG of Ser58, as well as the main chain N of Ser58. The α‐amino nitrogen of L‐Asp forms putative hydrogen bonds with the carboxylate groups of Asp57 and Asp88. Thus, conformation of the substrate and most of its interactions with active pocket residues are very similar to those described for typical class 1 ASNases. However, the interactions observed for the α‐amino group of the L‐Asp ligand are different in RrA and EcAII (Figure 8).

The hydroxyl oxygen of Thr16 is within H‐bonding distance from the OH atom of Tyr21, contributed by the second tight dimer of the RrA tetramer. The location and conformation of Tyr21 is similar to its equivalent in EcAII (Tyr25), although in the latter enzyme Tyr25 is contributed by the same protomer as Thr12. Whereas no ligands other than water molecules are observed in the active sites of some RrA structures presented here, conformations of the active site loops contributing Thr16 and Tyr21 are uniquely defined. However, disorder is often observed in the following loop (residues 58–60).

A distinctive feature of RrA is the presence of Lys19 within the lining of the substrate‐binding site. Given the high conservation of this residue it is likely that this feature is common to most scASNases. The amino group of Lys19 forms hydrogen bonds with OD2 of Asp57 and OD1 of Asn26, and it is in a close contact with the OH group of Tyr21. The Asn26 and Tyr21 residues are contributed by a protomer from the second tight dimer. There is no equivalent positively charged residue, except of catalytic Lys158, present in the vicinity of the active site in any characterized class 1 ASNase, making it interesting to evaluate the role of Lys19 in the enzymatic activity of RrA. As mentioned above, we probed the importance of this residue by mutating it to L‐Ala, L‐Glu, and L‐Gln.

### 2.8 Catalytic properties of RrA

The preliminary functional assays have revealed that, in terms of turnover numbers, the glutaminolytic activity of RrA(wt) is at least three orders of magnitude lower than its asparaginolytic activity. Therefore, RrA can be classified as an almost pure asparaginase. Measurements of the rate of L‐Asn hydrolysis by RrA(wt) in 14 solutions buffered at pH varying between 4.5 and 11.0, summarized in Figure S2, show that this enzyme is most active at pH 9.0. This finding aligns well with the results reported by Dobryakova et al. (Dobryakova et al., 2022) An analysis of the initial reaction velocities at various substrate concentrations (see Materials and methods) revealed that RrA‐catalyzed hydrolysis of L‐Asn follows the standard Michaelis–Menten model. The values of *K*_m_, determined for RrA(wt) at pH 7.4 and 9, are 3.6 ± 0.3 mM and 2.9 ± 0.3 mM, respectively. The associated turnover numbers (*k*_cat_) are 58.2 ± 3.0 s^−1^ and 57.1 ± 2.6 s^−1^, respectively. These values are also in good agreement with previously published results (Dobryakova et al., 2022). Although no cooperativity was detected, we observed strong substrate inhibition (Reed et al., 2010; Yoshino & Murakami, 2015) at concentrations of L‐Asn higher than 5 mM.

### 2.9 Enzymatic activity of RrA variants

For the mutated variants of RrA, our focus was on the catalytic activities expressed by approximate turnover numbers (due to low substrate concentration these are not “true” turnover numbers), determined under common conditions. Relative activities are presented in Table 3. Similar to the situation found in typical class 1 ASNases, mutation of the catalytic Lys residue (K158M in RrA) completely inactivates the enzyme (Lubkowski et al., 2020; Strzelczyk et al., 2020; Strzelczyk et al., 2023) (Table 3). Another mutated residue was Tyr21. Based on the previously published results for typical class 1 ASNases (Derst et al., 1994; Schalk et al., 2016), we tested two mutants, RrA(Y21A) and RrA(Y21F). Although both were less active than RrA(wt), RrA(Y21F) is somewhat more active than RrA(Y21A), which is opposite to the results obtained for typical class 1 ASNases. As reported in our earlier studies of class 1 ASNases (Lubkowski et al., 2020; Strzelczyk et al., 2020; Strzelczyk et al., 2023), the tyrosine equivalent to Tyr21 played a significant, although indirect role in catalysis, supporting proton transfer. In the case of EcAII, all mutations of the equivalent tyrosine, except Tyr → Lys, led to a decrease in activity by 10–100 times compared to EcAII(wt) (Derst et al., 1994; Lubkowski et al., 2020). While it is quite plausible that in RrA Tyr21 contributes to proton transfer from the primary nucleophile (Thr16), almost certainly its side chain also contributes to stabilization of the tetramer. In this role, substituting Tyr with Phe preserves this role better than the mutation Tyr → Ala. While the significance of tetramerization of scASNases (and in ASNases in general) is still unclear, the kinetic observation mentioned above may be helpful in a search for the correct interpretation. However, it appears that the role of Tyr21 in the enzymatic reaction in RrA is less important than for type II ASNases.

**TABLE 3:** Relative activities of RrA variants.

Variant	Relative activity (%)	Variant	Relative activity (%)
wt	100	Y21A	7.57
K19A	1.06	Y21F	28.95
K19E	0.1	K158M	0.0
K19Q	0.08		

Another residue that was mutated in order to assess its importance for the catalytic activity was Lys19. As mentioned earlier, this residue is highly conserved in scASNases but not in typical class 1 ASNases. Therefore, its role in RrA, and possibly in other scASNases, is quite intriguing. The kinetic data, even when reduced to approximate turnover numbers (Table 3), demonstrate that Lys19 plays an important role during catalysis and its mutation reduces the rate of catalysis by two or more orders of magnitude. As we indicated earlier, the amino group of this lysine is in close contact with several active site residues, including Tyr21, some of which (i.e., Asn26) are contributed by another tight dimer. It appears that Lys19 may play a comparable function to Tyr21, each being responsible for both stabilizing the tetramer and for supporting proton transfer. Further studies are necessary to explain the differences between the activities of the K19A, K19E, and K19Q variants. Possible implications of these findings are presented in the Discussion section.

### 2.10 Thermal stability of RrA

The stability of RrA was studied using the NanoDSF technique, which allows the measurement of three parameters characterizing a sample in solution: the temperature of aggregation (*T*_agg_), the temperature of unfolding (*T*_onset_), and the melting temperature (*T*_m_). Parameter *T*_agg_ is obtained from the measurement of the temperature dependence of light scattering. Its determination is possible only with the back‐reflection attachment. Table S1 shows that, in the case of RrA(wt), the aggregation propensity greatly depends on pH. At pH 5.0, the enzyme starts to aggregate at 42.3°C, whereas the same effect at pH 9.0 is observed at 74.1°C. Additionally, the maximum value of scattering within the studied temperature range is much higher at pH 5.0 (351.6 mAU) than at pH 9.0 (143.2 mAU). However, this difference is much smaller (330 vs. 250 mAU) in the presence of L‐Asp or L‐Glu. Aggregation occurs more rapidly at lower pH values (based on the first derivative of scattering, data not shown here) than at pH 9.0. At pH 5.0, *T*_agg_ increases significantly in the presence of either L‐Asp or L‐Glu (values of *T*_agg_ are 61.3 and 61.5°C, respectively). At pH 7.0, ligands have little effect on stability, at pH 9.0 either ligand destabilizes the structure. These findings are consistent with earlier results suggesting that only the protonated form of L‐Asp (or sometimes L‐Glu) binds stably to the ASNase active site (Lubkowski et al., 2020; Röhm & Van Etten, 1986). However, since L‐Gln is not hydrolyzed by RrA, stabilization provided by L‐Glu may be due to non‐productive substrate binding (Aghaiypour et al., 2001; Nguyen et al., 2016). Additional arguments for such an interpretation come from the results of attempted co‐crystallization of RrA with L‐Glu, with no ligand seen in the active site pocket (data not shown).

Two other parameters, *T*_onset_ and *T*_m_, indicating the beginning of unfolding and the melting temperature, respectively, are reported by changes in fluorescence at 330 nm, 350 nm, or the 350/330 ratio. While the values of *T*_onset_ are usually easily obtainable, sometimes a complexity of thermograms and their derivatives obstructs unequivocal interpretation. RrA is devoid of tryptophan residues that typically create a strong and reliable signal in NanoDSF experiments (Kim et al., 2021). Only three tyrosine residues are present in the RrA protomer, two of which are solvent‐exposed in the native state and do not contribute significantly to the NanoDSF temperature‐induced fluorescence changes. Nevertheless, the trends observed with an increase in temperature are similar to those described above for *T*_agg_. However, in the absence of stabilizing ligands, both *T*_onset_ and *T*_m_ are much lower than *T*_agg_ at pH 9.0. A plausible explanation is a change in the quaternary state of the protein, i.e. dissociation of tetramers to dimers. As mentioned above, the environment of just one tyrosine in a protomer of RrA changes upon either unfolding or dissociation of the tetramer.

## 3 DISCUSSION

### 3.1 Classification and cellular location of L‐asparaginases

Class 1 ASNases are defined as enzymes composed of approximately 300+ amino acids. They are divided into cytosolic type I enzymes, with millimolar affinity for L‐Asn, and periplasmic type II enzymes, having low‐to‐modest micromolar affinity for this substrate. However, data shown here indicate that this classification may require an update and extension. On a pan‐genomic scale, only a subset of the enzymes in the type II ASNase group possess a predicted signal peptidase (SPase) I secretion signal, while many are predicted to be cytosolic or have an SPase II signal peptide that suggests membrane anchoring. Thus, enzymes characterized as type II ASNases based on their sequence and/or low *K*_m_ for substrate binding should be classified differently. The putative membrane‐anchored enzymes do not fit exactly into their assigned category as well. We propose that sequence similarity and phylogeny should serve as the criteria for differentiating between type II secreted (IIs), cytosolic (IIc), and membrane‐anchored (IIm) family members.

### 3.2 A phylogenetically distinct family of new scASNases

In this work, we have demonstrated that RrA can be considered as a representative of a whole family of scASNases present in a wide variety of bacteria (and eukarya, as will be shown in a separate study). The discovery of scASNases described here complicates the current classification of ASNases. On the level of nucleotide or protein sequence, scASNases resemble N‐terminal domains of typical type I ASNases. Furthermore, both type I ASNases and scASNases have low affinity for L‐Asn. Nevertheless, CLANS (Frickey & Lupas, 2004) analysis reveals that scASNases occupy a distinct region of sequence space, even when just the first 150 amino acids are compared in order to eliminate length‐dependent clustering. Therefore, we suggest that scASNases should be classified as a separate subgroup, i.e. as a distinct family among the class 1 enzymes.

### 3.3 Unique structural and biochemical features of scASNases

Accounting for the high level of sequence conservation among scASNases, we suggest that most of the characteristics observed for RrA are likely shared by an entire family of these enzymes. Unlike typical class 1 ASNases, protomers of scASNases consist of approximately 160–185 amino acid residues (172 in RrA). Their amino acid sequences and structures of protomers are similar to those of the N‐terminal domains of class 1 ASNases. Local differences resulting from the shorter length of the polypeptide chains in scASNases were described in the Results section. Catalytically, RrA is most similar to type 1 ASNases, demonstrating that, despite their small size, scASNases are capable of hydrolyzing L‐Asn. Two other observations are quite remarkable. Whereas in both typical class 1 ASNases and scASNases a dimer is the smallest structural unit necessary for supporting catalytic activity, the architecture of such dimers in each of these two families is completely different. However, despite this important structural difference, the structure and composition of the active site pocket in scASNases is strikingly similar to that found in typical class 1 enzymes and most residues in the catalytic sites are the same.

### 3.4 scASNase homotetramers

It is important to note that RrA forms homotetramers in solution even at low picomolar concentrations, as demonstrated by MP. These homotetramers result from dimerization of tight homodimers. At present, the role of tetramerization in ASNases (including scASNases) remains unclear. However, tetramerization found in scASNases leads to interactions that are unique to this subgroup of asparaginases. One particularly intriguing interaction is observed between Lys19(NZ) (a residue unique to scASNases where it is highly conserved, see Supplementary sequence alignment S2) and Tyr21(OH), an equivalent of which is believed to play a significant role in proton transfer during catalysis in class 1 ASNases. In the catalytic process, the tyrosine side chain may provide the same functionality in both ASNase groups, namely create a path for proton transfer from Thr16 to Asp88/Lys158. In RrA, and possibly other scASNases, both Tyr21 and Lys19 may likely play additional roles other than assisting catalysis, by contributing to stabilization of the homotetramer.

### 3.5 Catalytic mechanism

Catalysis by typical class 1 asparaginases involves formation of a covalent acyl‐enzyme intermediate (Derst et al., 1994). In the case of RrA, an acyl‐enzyme intermediate, generally considered as the best identifier of the primary nucleophile, has not yet been observed. However, that does not mean that both families differ in terms of the mechanism of catalysis. First, to trap an acyl‐enzyme intermediate, the acylation (1st) step needs to be much faster than the deacylation (2nd) step, a condition not necessarily true in the case of every ASNase. Furthermore, a substrate molecule must bind productively to the active site for the catalytic reaction to proceed, which in the case of L‐Asp as the substrate requires acidic pH. However, at pH <6, RrA is unstable and this effect greatly hinders crystallization efforts under these conditions, preventing experimental verification of the mechanism.

Without direct evidence for the reaction mechanism resembling that for class 1 ASNases, helpful clues may be obtained from analyzing conservation of active site residues. The reaction mechanism for class 1 asparaginases, recently described in detail (Lubkowski et al., 2020), posits involvement of two atypical catalytic triads (Dodson & Wlodawer, 1998). This mechanism also requires the presence of a serine residue (Ser58 in EcAII) as a crucial component for substrate binding (Lubkowski et al., 2020). With the exception of Glu283, which lies beyond the region present in scASNases, all catalytic residues are conserved or functionally conserved in RrA (i.e. Tyr21 in RrA is equivalent to Tyr25 in EcAII) (Figure 8). Except for the lysine located near the C terminus of the scASNases (Lys158 in RrA, equivalent to Lys162 in EcAII), all residues important for catalysis are also quite well conserved among the scASNases (Figure 4). Therefore, we postulate that major steps of the enzymatic reaction in scASNases are equivalent or very similar to those described previously for class 1 ASNases.

### 3.6 Perspectives

With the length of a protomer of scASNases being less than half of its counterpart in typical class 1 ASNases, these enzymes may present fewer sites likely to elicit an immune reaction responsible for frequent side effects of therapy involving ASNases in current clinical practice. However, a major barrier for therapeutic use of scASNases is their millimolar substrate affinity. As scASNases are more similar to the type I than to type II asparaginases (Figure 2), it is likely that the lower substrate affinity is a feature that is shared among all members of this group of enzymes. Since problems caused by low substrate affinity were frequently mentioned when new type I ASNases were described (Ashok et al., 2019; Chi et al., 2023; Guo et al., 2017), this limitation is also present for scASNases. Although there have been no successful attempts to reduce the *K*_m_ of typical type I ASNases that have been reported, such experiments have not been performed with scASNases. Whether such engineering would be possible remains to be seen.

## 4 MATERIALS AND METHODS

### 4.1 Analysis of gene sequences

The nucleotide sequences of the entire bacterial genomes (3860 GenBank files), which represent 1554 distinct species, were downloaded from the EMBL microbial collection. These sequences are accessible at http://www.ebi.ac.uk/ena/browser/api/embl.

### 4.2 Protein cloning, expression, and purification

The synthetic gene of RrA (GenBank QXG80441.1), incorporating an additional N‐terminal sequence corresponding to the tobacco etch virus (TEV) protease cleavage site and with codons optimized for expression in *E. coli*, was procured from BioBasic (Markham, Ontario, Canada). The gene was subsequently cloned into the pET15b vector, resulting in the open reading frame encoding His_6_tag‐TEVPR_cleavage_site_‐RrA. Our RrA gene differs at one position from the sequence initially reported by Pokrovskaya et al. (Pokrovskaya et al., 2012), with Lys occupying position 149 instead of Glu. This variation appears to be a natural occurrence, as Lys149 is observed in several sequences listed in the NCBI database, including the one depicted in figure 10 of Pokrovskaya et al. (Pokrovskaya et al., 2015).

All plasmids encoding mutated variants of RrA were created using the QuikChange protocol (Agilent, USA), and the corresponding proteins were expressed in BL21(DE3) RIPL cells. Cell cultures (1 L) were cultivated at 37°C in a shaker‐incubator with an agitation rate of 250 rpm in the Luria‐Bertani broth supplemented with ampicillin (100 μg/mL) and chloramphenicol (30 μg/mL) until the optical density, measured at 600 nm, reached 0.5. Subsequently, protein expression was induced with isopropyl β‐D‐1‐thiogalactopyranoside (0.5 mM). The temperature was then reduced to 18°C and cell growth continued overnight.

On the following morning the cell pellet was harvested, suspended in 50 mM Tris, 0.5 M NaCl (pH 8), and lysed using a microfluidizer. The resulting lysate was clarified via centrifugation at 15,000 rpm at 4°C and filtration using a 0.45 μm filter (Nalgene, ThermoFisher Scientific, Waltham, MA). The cleared filtrate was applied on a 5 mL HisTrap HP column (Cytiva, Marlborough, MA), and the elution was carried out using 50 mM Tris, 0.5 M NaCl, and 250 mM imidazole, pH 8. The eluted fractions were combined, supplemented with 5 mg of TEV protease, and dialyzed overnight against 50 mM Tris, 0.5 M NaCl, pH 8, at 4°C.

The resultant protein solution was once again subjected to a 5 mL HisTrap HP column, with the flow‐through fractions collected, pooled, concentrated to 5 mL, and subsequently applied to a Superdex 75 16/60 size exclusion column (Cytiva, Marlborough, MA). The column was pre‐equilibrated with the standard buffer. Fractions containing RrA were pooled, concentrated to 20 mg/mL, and stored in a –80°C freezer.

### 4.3 Crystallization, collection of x‐ray data, structure solution, and refinement

In all experiments, proteins (19–21 mg/mL) were dissolved in the standard buffer. All crystallizations were conducted using hanging drops created from equal volumes of protein and precipitant solutions at temperature 293 K. Crystallization conditions and cryoprotectant solutions are described in Tables S2A through S2D. Diffraction data were collected at the Advanced Photon Source, Argonne National Laboratory (Argonne, IL, USA), at beamline 22‐ID. All measurements were performed at 100 K. Subsequent image processing and scaling were carried out with HKL3000 (Minor et al., 2006).

For solving each structure, molecular replacement was independently performed using the program Phaser (McCoy et al., 2007). The predicted RrA monomer structure generated by the AlphaFold2 program (Jumper et al., 2021) was used as the template. Clearly identifiable solutions were positioned within the unit cell based on calculations from the Achesym server (Kowiel et al., 2014). These solutions were subjected to rigid‐body refinement at a resolution of 2.5 Å using the program Refmac5 (Murshudov et al., 2011). This step was followed by refinement of positions and atomic displacement parameters (B‐factors) for non‐hydrogen atoms. Subsequently, resolution was extended to the limits of experimental data.

Models were examined and adjusted using the program Coot (Emsley & Cowtan, 2004) and solvent molecules and other ligands were incorporated. The final models were evaluated using the MolProbity server (Chen et al., 2010). Detailed information regarding data collection and processing statistics can be found in Table S3.

### 4.4 Dynamic light scattering measurements

Prior to measurements, solutions of RrA were prepared at various concentrations in the standard buffer. These solutions were then subjected to centrifugation at 14,000 rpm (approximately 20,000 × *g*) for 20 min to remove any aggregates. Measurements were carried out using the DynaPro Nanostar instrument (Wyatt, Santa Barbara, CA) at 20°C. The results were subsequently analyzed using the manufacturer's program Dynamics (version 7.10.1.21).

Each experiment involved 100 scans, 1 s each. The data presented here are the mean values derived from these scans. It is important to note that proteins were assumed to have a globular structure.

### 4.5 Mass photometry measurements

The mass distributions of RrA samples were assessed using a Refeyn TwoMP instrument (Refeyn, Oxford, UK). The measurements adhered to the standard protocol as outlined in Wu & Piszczek (2021). In short, sample chambers were assembled by positioning silicon gasket wells onto 24 mm × 50 mm coverslips. Initially, the sample wells were loaded with predetermined volumes of the standard buffer, having been passed through a 0.22 μm syringe filter. Solutions of RrA were clarified through centrifugation, with the enzyme concentrations determined based on the absorbance at 280 nm and on theoretical extinction coefficient at this wavelength. Data were gathered at two distinct concentrations, 25 and 65 nM, in the standard buffer. A one‐minute video was recorded employing the AcquireMP software and subsequent analysis was performed using DiscoverMP (Refeyn, Oxford, UK). Before each RrA measurement, a mixture of well‐characterized proteins (unstained protein ladder, LC0725, ThermoFisher Scientific, Waltham, MA) was measured under identical conditions. This measurement was used for reliable calibration of molecular weights.

### 4.6 Nano differential scanning fluorimetry (NanoDSF)

Thermostability of the enzymes was evaluated using the NanoDSF device, Prometheus NT.48 (NanoTemper Technologies, Watertown, MA). The device was equipped with aggregation optics. This experiment enabled concurrent recording of fluorescence emission spectra at 330 nm and 350 nm, as well as the intensity of back‐reflection at 385 nm.

The 350/330 nm emission ratio was utilized to determine the onset of unfolding (*T*_onset_) and the melting temperature (*T*_m_). Meanwhile, alterations in scattering profiles, as observed through back‐reflection, facilitated identification of the initiation of aggregation (*T*_agg_). For each enzyme variant, 10 μL samples were employed, with concentrations near 5 mg/mL. The samples were dissolved in one of three buffers: 0.1 M Na‐citrate pH 5, 0.1 M HEPES pH 7, and 0.1 M Tris–HCl (pH 8.5), all of which were supplemented with 0.2 M NaCl, and placed in quartz capillaries.

Measurements for each RrA variant were taken in one of the three buffers listed above in the absence of a ligand. Additionally, experiments were also performed after addition of 20 mM L‐Asp or 20 mM L‐Glu. Temperature scans were performed using a linear rate (1°C/min) with a range from 20 to 80°C. The fluorescence and aggregation scans were subsequently analyzed using the proprietary software provided by the manufacturer. In each condition, the analysis was based on the averages derived from three replicates.

### 4.7 Catalytic rate of RrA at different pH values

Experiments were conducted at 20°C using 0.1 M buffers supplemented with 0.2 M NaCl, and calibrated to pH values ranging between 4.5 and 11. The buffers employed were as follows: citrate (pH 4.5 and 5), MES (pH 5.5, 6, and 6.5), cacodylate (pH 7), HEPES (pH 7.5), Bis‐Tris Propane (pH 8 and 9), Tris (pH 8.5), CHES (pH 9.5), and CAPS (pH 10, 10.5, and 11).

Upon a 2‐min incubation period, each reaction was halted by the addition of 12% trichloroacetic acid (TCA), and the concentration of ammonia was promptly determined with the use of Nessler's reagent. In the case of RrA(wt), the enzyme and L‐Asn (or L‐Gln) concentrations within the reaction mixture were set at 500 nM and 2.4 mM, respectively. Each data point was obtained in at least four separate measurements.

### 4.8 Kinetic properties

Two sets of experiments were conducted for RrA(wt). The first set aimed to determine the *K*_m_ and *V*_max_ (or *k*_cat_) values for the hydrolysis of L‐Asn at two distinct pH values: 7.4 (using PBS buffer: 137 mM NaCl, 2.7 mM KCl, 8 mM Na_2_HPO_4_, and 2 mM KH_2_PO_4_) and 9.0 (using 50 mM K_2_CO_3_/KHCO_3_ buffer with 150 mM NaCl). In these experiments, concentrations of RrA(wt) and reaction times (prior to quenching with 12% TCA) were set at 282 nM and 60 s, respectively, at pH 7.4, and 72 nM and 120 s at pH 9.0. Both sets of experiments were conducted using L‐Asn at various concentrations ranging from 95 μM to 67 mM.

The second set of experiments, carried out for both RrA(wt) and for all mutants, was conducted in the PBS buffer (pH 7.4). Depending on the catalytic activity of the tested variants, the assays were executed with one of two enzyme concentrations: 200 nM or 2 μM, and reaction times of either 120 or 600 s. In all cases, substrate concentration remained constant at 3.8 mM. For RrA(wt), the assays were performed using both L‐Asn and L‐Gln as substrates. While these experiments do not yield exact kinetic parameters, they enable determination of relative catalytic rates of mutants compared to the wild‐type enzyme under the specified conditions.

Both sets of experiments were conducted at a temperature of 20°C. After potential precipitates were removed through centrifugation and subsequent nesslerization, the progress of catalytic reactions was monitored by measuring the optical absorbance at 480 nm (A_480 nm_). This absorbance is linked to the formation of ammonia. All measurements were performed using 1 cm cuvettes in an Eppendorf BioSpectrometer Kinetic (Eppendorf AG, Hamburg, Germany). The molar absorption coefficient of the Nessler's product, *ε*_480 nm_ = 1302 ± 20 M^−1^ cm^−1^, was determined during a calibration experiment. Blank solutions of L‐Asn lacking enzyme were employed to compensate for non‐enzymatic hydrolysis of L‐asparagine. The reported values represent averages from at least four experiments. Any variant characterized by a turnover number at pH 7.4 that was at least three orders of magnitude lower than that of RrA(wt) was deemed inactive. Data were processed using the Microsoft Excel program, and double‐reciprocal, Lineweaver–Burk plots were employed to deduce values of *K*_m_ and *V*_max_ (*k*_cat_).

## AUTHOR CONTRIBUTIONS

**Di Zhang:** Investigation; data curation. **Honorata Czapinska:** Investigation; data curation. **Matthias Bochtler:** Conceptualization; supervision; investigation; writing – original draft; funding acquisition; validation. **Alexander Wlodawer:** Supervision; conceptualization; writing – original draft; funding acquisition; validation. **Jacek Lubkowski:** Conceptualization; supervision; writing – review and editing; writing – original draft; project administration; validation.

## Supporting information


**Data S1.** Supporting Information.

**Data S2.** Supplementary sequence alignment.

**Data S3.** Genome accesssions.

## Data Availability

Crystallographic data for the structures reported in this article have been deposited at the Protein Data Bank with the accession numbers 8uou, 8uoo, 8up6, 8uow, 8up9, 8uor, 8up7, 8up8, 8up3, and 8upc.
