# Supplementary material for: RrA, an enzyme from Rhodospirillum rubrum, is a prototype of a new family of short‐chain L‐asparaginases
Source: Protein Sci. 2024 Mar 19;33(4):e4920. doi: 10.1002/pro.4920 (PMC10949315; doi:10.1002/pro.4920)
Supplement: Supplementary file 1 — Data S1. Supporting Information. [file PRO-33-e4920-s003.pdf]

Supplementary information

RrA – a prototype of a new family of short-chain L-asparaginases

Di Zhang[1], Honorata Czapinska[2,3], Matthias Bochtler[2,3,&], Alexander Wlodawer[1,&], Jacek Lubkowski[1,&,*]

Affiliations:

[1] Center for Structural Biology, National Cancer Institute, Frederick, MD, USA

[2] International Institute of Molecular and Cell Biology, Warsaw, Poland

[3] Polish Academy of Sciences, Institute of Biochemistry and Biophysics, Warsaw, Poland

[&] lubkowsj@mail.nih.gov, wlodawer@nih.gov, mbochtler@iimcb.gov.pl

[*] Corresponding author

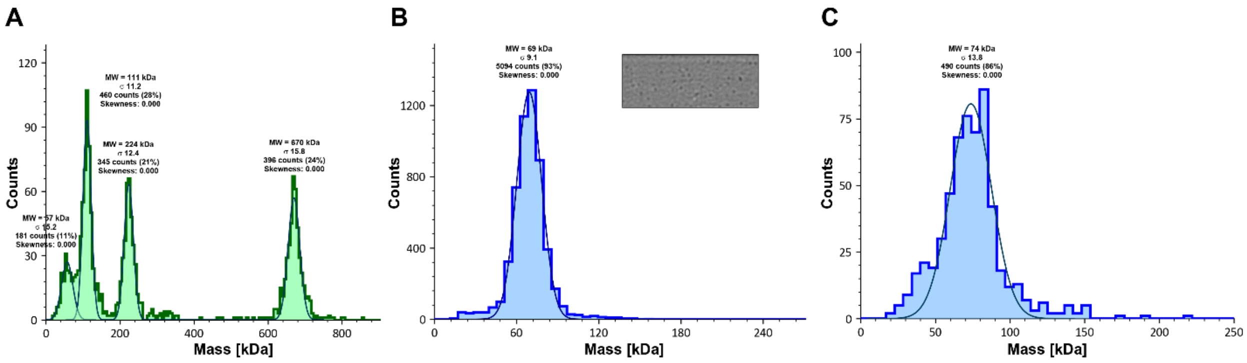

Figure S1. Determination of mass distributions by MP. (A) Histograms of the masses associated with single molecule surface absorption events for selected protein standards. **(B-C)** Histograms of masses for RrA(wt) at two concentrations, 65 nM (B) and 25 nM (C). The molecular weights of all peaks were calibrated against the proteins with well-defined MWs (shown in panel A). In each panel, dominant peaks are marked with corresponding MW values, the standard deviation σ, number (and percentage) of successful counts from experimental images, and skewness indicator of peak asymmetry. The insert in panel B shows the ratiometric view of protein landing events recorded for RrA(wt).

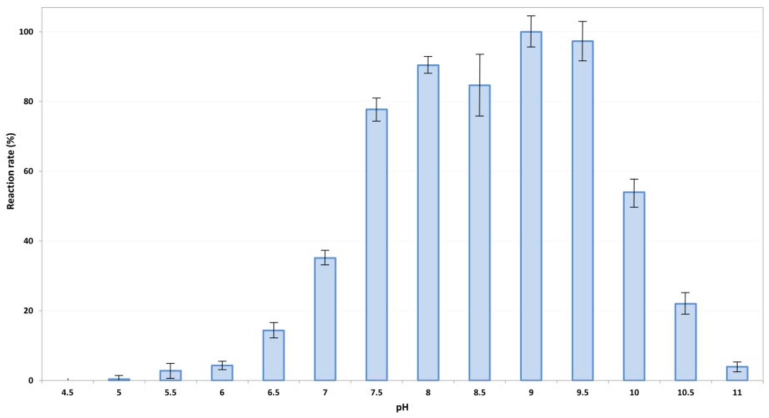

Figure S2. Activity of RrA at different pH. The catalytic activity of RrA was measured at the pH range between 4.5 and 11 as described in Materials and methods. The enzyme is most active at pH 9.

Table S1. Thermal stability of RrA and its mutated variants[a]

**RrA(wt)**									
**Ligand**	None			L-Asp			L-Glu		
**pH**	5	7	9	5	7	9	5	7	9
**T_{agg} (°C)[b]**	32.5	54.0	56.8	47.5	48.5	57.5	48.0	47.9	57.2
**T_{onset} (°C)**	33.7	52.9	54.2	46.1	51.7	53.6	49.9	46.4	53.5
**T_m (°C)**	41.7	56.2	57.6	61.7	54.4	55.3[c]<br>62.1	62.2	49.3<br>55.8	55.8<br>60.8
**RrA(Y21F)**									
**T_{agg} (°C)[b]**	48.3	54.6	58.0	45.6	47.7	57.2	45.8	47.7	56.8
**T_{onset} (°C)**	48.3	53.5	52.7	45.9	48.5	52.9	46.0	48.5	52.1
**T_m (°C)**	52.8	57.3	57.3	51.4	52.6	57.2	51.6	52.8	56.8
**RrA(Y21A)**									
**T_{agg} (°C)[b]**	40.1	51.1	62.2	32.4	41.5	52.0	33.3	42.2	51.1
**T_{onset} (°C)**	38.8	45.9	44.6	32.5	40.5	44.3	32.9	41.2	44.5
**T_m (°C)**	45.0	52.0	50.6	41.2	46.1	50.6	41.4	46.8	50.4
**RrA(K19A)**									
**T_{agg} (°C)[b]**	43.7	54.4	58.4	37.5	45.2	56.7	36.4	43.7	59.3
**T_{onset} (°C)**	30.0	49.3	51.7	36.5	45.4	51.2	36.3	43.4	50.7
**T_m (°C)**	42.3<br>49.6<br>60.7	54.6<br>56.8	54.6	43.1	50.2	54.6	43.3	48.7	54.2
**RrA(K19Q)**									
**T_{agg} (°C)[b]**	49.3	54.3	66.5	44.9	46.5	63.5	47.3	46.0	n.d.
**T_{onset} (°C)**	54.6	49.5	49.0	53.5	49.1	47.2	57.0	44.1	48.5
**T_m (°C)**	63.5	51.4<br>57.2	52.6<br>57.5	61.8	52.4	52.5<br>57.4	62.7	47.3<br>51.8	52.2<br>57.1
**RrA(K19E)**									
**T_{agg} (°C)[b]**	46.7	55.2	50.1	43.0	48.7	n.d.	44.1	47.0	37.8
**T_{onset} (°C)**	48.2	50.4	47.9	46.1	49.4	48.4	46.9	48.3	48.0
**T_m (°C)**	51.2<br>61.4	54.5	53.3	40.4<br>49.2<br>61.2	52.6	53.4	49.0<br>62.4	51.5	53.2
**RrA(K158M)**									
**T_{agg} (°C)[b]**	57.0	62.4	63.3	54.6	54.3	62.9	55.5	55.7	62.8
**T_{onset} (°C)**	57.9	61.2	60.0	66.9	58.0	60.0	66.5	51.5	59.9
**T_m (°C)**	72.0	62.3<br>67.8	62.6	71.5	62.0	62.8	71.6	54.7<br>63.1	62.6

[a]All values reported here represent averages of at least three independent measurements
[b]These values were obtained from the scattering vs. temperature graphs
[c]For these runs, two inflection points have been observed on ratio (see graphs).

Table S2. Crystallization conditions and cryo-protecting solutions

**RrA(wt)**	***Reservoir***	0.2 M $MgCl_2$, 0.1 M HEPES pH 7.5, 30% v/v PEG400
	Cryo-protectant	Reservoir solution enriched with 15% v/v glycerol
**RrA(wt)/L-Asp**	***Reservoir***	0.2 M $MgCl_2$, 0.1 M HEPES pH 7.5, 30% v/v PEG400, 20 mM L-Asp
	Cryo-protectant	Reservoir solution enriched with 15% v/v glycerol
**RrA(K19A)**	0.12 M Alcohols; 0.1 M Buffer System 3; pH 8.5; 30% v/v Precipitant Mix 1 (Molecular Dimensions)[1,2]	
**RrA(K19A)/L-Asp**	0.12 M Monosaccharides; 0.1 M Buffer System 3; pH 8.5; 30% v/v Precipitant Mix 1 (Molecular Dimensions) + 20 mM L-Asp[1,2]	
**RrA(K19Q)**	0.09 M NPS; 0.1 M Buffer System 3; pH 8.5; 30% v/v Precipitant Mix 1 (Molecular Dimensions)[1,2]	
**RrA(K19E)**	0.06 M Divalents; 0.1 M Buffer System 1; pH 6.5; 30% v/v Precipitant Mix 2 (Molecular Dimensions)[1,2]	
**RrA(Y21A)**	0.6 % Antibiotics mix; 0.1 M Buffer System 2, pH7.5; 37.5 % Precipitant Mix 4 (Molecular Dimensions)[1,2]	
**RrA(Y21F)**	0.06 M Divalents; 0.1 M Buffer System 1; pH 6.5; 30% v/v Precipitant Mix 2 (Molecular Dimensions)[1,2]	
**RrA(Y21F)/L-Asp**	0.12 M Monosaccharides; 0.1 M Buffer System 3; pH 8.5; 30% v/v Precipitant Mix 1 (Molecular Dimensions) + 20 mM L-Asp[1,2]	
**RrA(K158M)**	0.06 M Halogens; 0.1 M Buffer System 3; pH 8.5; 37.5% v/v Precipitant Mix 4 (Molecular Dimensions)[1,2]	

[1]Does not require separate cryo-protectant.

[2]For detailed description see https://www. moleculardimensions.com/products/all-crystallization-screens.

Mixes of precipitants used in Morpheus

Mix name	Composition
Precipitant Mix 1	40% v/v PEG 500 MME; 20% w/v PEG 20000
Precipitant Mix 2	40% v/v Ethylene glycol; 20% w/v PEG 8000
Precipitant Mix 4	25% v/v MPD; 25% PEG 1000; 25% w/v PEG 3350

Buffer systems used in Morpheus

Mix Name	Concentration	pH@20C	Composition
Buffer System 1	1.0 M	6.5	Imidazole; MES monohydrate (acid)
Buffer System 2	1.0 M	7.5	Sodium HEPES; MOPS (acid)
Buffer System 3	1.0 M	8.5	Tris (base); BICINE

Mixes of additives used in Morpheus

Mix name	Composition
Alcohols	0.2 M 1,6-Hexanediol; 0.2 M 1-Butanol; 0.2 M 1,2-Propanediol; 0.2 M 2-Propanol; 0.2 M 1,4-Butanediol; 0.2 M 1,3-Propanediol
Antibiotics	1% w/v Ampicillin sodium salt; 1% w/v Apramycin sulfate salt; 1% w/v Bacitracin; 1% w/v Dihydrostreptomycin sesquisulfate; 1% w/v Gentamicin sulfate; 1% w/v Spectinomycin dihydrochloride pentahydrate
Divalents	0.3 M Magnesium chloride hexahydrate; 0.3 M Calcium chloride dihydrate
Halogens	0.3 M Sodium fluoride; 0.3 M Sodium bromide; 0.3 M Sodium iodide
NPS (Nitrate Phosphate Sulfate)	0.3 M Sodium nitrate; 0.3 M Sodium phosphate dibasic; 0.3 M Ammonium sulfate
Monosaccharides	0.2 M D-Glucose; 0.2 M D-Mannose; 0.2 M D-Galatctose; 0.2 M L-Fucose; 0.2 M D-Xylose; 0.2 M N-Acetyl-D-Glucosamine

Table S3. Statistics of X-ray data collection and refinement.

Structure	RrA(wt) + L-Asp	RrA(wt)	RrA(K19A) + L-Asp	RrA(K19A)	RrA(K19Q)
Space group	$P2_12_12$	$P2_12_12$	$P2_12_12_1$	$P2_12_12$	$P2_12_12$
Unit cell parameters (Å)	71.46, 76.13, 55.36	72.18, 76.96, 57.82	72.05, 77.33, 115.03	72.11, 77.36, 58.20	72.57, 77.37, 57.74
**Data Collection Statistics**					
Completeness (%)*<br>Redundancy	98.1 (85.3)<br>10.9 (4.7)	95.2 (92.9)<br>10.0 (6.6)	99.6 (99.8)<br>12.9 (11.9)	95.0 (93.1)<br>11.1 (7.2)	98.6 (99.6)<br>6.2 (5.8)
I/σ(I)	27.4 (1.6)	21.4 (1.6)	32.9 (3.7)	21.9 (3.3)	15.5 (2.5)
Unique reflections	65,882 (2810)	37,670 (1784)	71,088 (3496)	34,698	55,366 (2777)
High resolution shell (Å)	1.35 – 1.37	1.65 – 1.69	1.70 – 1.73	1.70 – 1.73	1.50 – 1.47
R-linear<br>R-square<br>R_{pim}<br>$CC_{1/2}$	0.073 (0.846)<br>0.062 (0.762)<br>0.023 (0.413)<br>0.994 (0.647)	0.098 (0.986)<br>0.064 (0.888)<br>0.033 (0.347)<br>0.996 (0.719)	0.068 (0.753)<br>0.045 (0.552)<br>0.020 (0.222)<br>1.000 (0.947)	0.116 (1.091)<br>0.072 (0.915)<br>0.036 (0.377)<br>0.993 (0.767)	0.088 (0.779)<br>0.086 (0.787)<br>0.040 (0.355)<br>0.995 (0.763)
**Refinement Statistics**					
Resolution (Å)	1.35-38.0<br>(1.35-1.39)	1.65-39.0<br>(1.65-1.69)	1.70-38.9<br>(1.70-1.745)	1.70-39.1<br>(1.70-1.745)	1.47-39.0<br>(1.47-1.50)
No. reflections:<br>refinement<br>validation	 60,006<br>1986	 31,966<br>1708	 69,304<br>1431	 31,204<br>1584	 52,680<br>1345
Protein chains in a.u.[@]	(A, B)	(A, B)	[(A,B),(C,D)]	(A, B)	(A, B)
No. of non-H atoms:<br>total<br>water<br>ligands	 2878<br>351<br>22	 2563<br>236<br>13	 5340<br>385<br>66	 2647<br>230<br>1	 2839<br>297<br>35
Type of ligand	non-covalent complex with L-Asp, Cl^-	Cl^-, GOL	non-covalent complex with L-Asp, PEG	Cl^-	Cl^-, EDO, SO_4^{-2}
ADP model[&]	anisotropic	anisotropic	isotropic	isotropic	anisotropic
Average ADP ($Å^2$): total<br>protein<br>water	19.3<br>17.7<br>31.0	19.0<br>18.4<br>24.6	19.1<br>18.6<br>22.7	22.7<br>22.2<br>27.6	22.5<br>21.2<br>33.2
R-factor	0.125 (0.251)	0.154 (0.185)	0.182 (0.230)	0.200 (0.282)	0.128 (0.193)
R_{free}	0.170 (0.301)	0.221 (0.325)	0.205 (0.280)	0.233 (0.313)	0.169 (0.253)
Rmsd values:<br>bond lengths (Å)<br>bond angles (˚)	 0.017<br>2.116	 0.014<br>1.689	 0.012<br>1.776	 0.011<br>1.586	 0.015<br>1.969
Estimated coordinate error (Å)[#]	0.030	0.061	0.067	0.081	0.038
PDB ID	8uou	8uoo	8up6	8uow	8up9

Structure	RrA(K19E)	RrA(Y21A)	RrA(Y21F) + L-Asp	RrA(Y21F)	RrA(K158M)
Space group	$P2_12_12$	$P2_12_12_1$	$P2_12_12_1$	$C2$	$P2_12_12$
Unit cell parameters (Å,˚)	72.58, 77.44, 57.76	71.83, 77.12, 114.85	72.14, 77.64, 115.18	245.64, 63.18, 92.97<br>90, 103.42, 90	72.15, 77.51, 58.05
**Data Collection Statistics**					
Completeness (%)*<br>Redundancy	96.0 (95.1)<br>6.0 (5.9)	98.5 (97.3)<br>4.7 (4.6)	99.0<br>100.0	97.1 (97.0)<br>4.1 (3.9)	97.0 (72.8)<br>11.0 (3.9)
I/σ(I)	42.3 (2.2)	22.3 (4.7)	23.0 (4.6)	18.5 (2.1)	27.5 (1.9)
Unique reflections	56,037 (2717)	84,496 (4101)	50,786 (2496)	132,560 (6556)	99,988 (3707)
High resolution shell	1.45 – 1.48	1.60 – 1.63	1.90 – 1.93	1.75 (1.78)	1.29 - 1.20
R-linear<br>R-square<br>R_{pim}<br>$CC_{1/2}$	0.044 (1.009)<br>0.025 (0.873)<br>0.019 (0.444)<br>0.999 (0.757)	0.051 (0.326)<br>0.055 (0.268)<br>0.025 (0.164)<br>0.991 (0.948)	0.079 (0.544)<br>0.061 (0.499)<br>0.026 (0.156)<br>1.000 (0.947)	0.080 (0.994)<br>0.062 (1.084)<br>0.043 (0.560)<br>0.994 (0.627)	0.078 (0.695)<br>0.080 (0.989)<br>0.023 (0.351)<br>0.998 (0.762)
**Refinement Statistics**					
Resolution (Å)	1.45-39.1<br>(1.45-1.49)	1.59-38.8<br>(1.59-1.63)	1.90-39.0<br>(1.90-1.95)	1.76-39.4<br>(1.76-1.81)	1.20-39.1<br>(1.20-1.25)
No. reflections:<br>refinement<br>validation	 53,949<br>1455	 81,672<br>2582	 49,360<br>1239	 127,611<br>1657	 91,411<br>1843
Protein chains in a.u.[@]	(A, B)	[(A,B),(C,D)]	(A, B), (C,D)	[(A, B), (C,D)]<br>[(E,F), (G,H)]	(A, B)
No. of non-H atoms:<br>total<br>water<br>ligands	 2837<br>306<br>26	 5401<br>616<br>32	 5341<br>382<br>79	 10,920<br>1080<br>120	 2856<br>345<br>14
Type of ligand	Cl^-, EDO	EPE	non-covalent complex with L-Asp, PEG	Cl^-, EDO,	Cl^-, GOL
ADP model[&]	anisotropic	isotropic	isotropic	isotropic	anisotropic
Average ADP ($Å^2$): total<br>protein<br>water	22.2<br>20.8<br>32.8	19.8<br>18.4<br>30.7	26.8<br>26.4<br>29.5	24.1<br>23.0<br>33.2	20.9<br>19.4<br>31.1
R-factor	0.123 (0.210)	0.187 (0.221)	0.179 (0.193)	0.165 (0.219)	0.140 (0.391)
R_{free}	0.166 (0.289)	0.221 (0.273)	0.225 (0.270)	0.216 (0.209)	0.164 (0.430)
Rmsd values:<br>bond lengths (Å)<br>bond angles (˚)	 0.017<br>2.057	 0.013<br>1.874	 0.011<br>1.687	 0.012<br>1.741	 0.013<br>1.802
Est. coordinate error (Å)[#]	0.033	0.054	0.091	0.071	0.017
PDB ID	8uor	8up7	8up8	8up3	8upc

*Values shown in parentheses correspond to the highest resolution shell. [@]Tight dimers in parentheses, tetramers in brackets. EDO-1,2 ethanediol; GOL-glycerol; PEG-polyethylene glycol; EPE-Hepes; [&]ADP – atomic displacement parameters. [#]Error of coordinates was estimated based on maximum likelihood.
