# Supplementary material for: RrA, an enzyme from Rhodospirillum rubrum, is a prototype of a new family of short‐chain L‐asparaginases
Source: Protein Sci. 2024 Mar 19;33(4):e4920. doi: 10.1002/pro.4920 (PMC10949315; doi:10.1002/pro.4920)
Supplement: Supplementary file 2 — Data S2. Supplementary sequence alignment. [file PRO-33-e4920-s002.pdf]

Sequence alignment of non-redundant scASNases using the program Clustal ver. 2.1. See caption under the alignment for explanation.

```
CP006890.1_[255482_256010]_+_       MKKQFMPFITFITALLFIAAGCSQSPQAKETPPRMAAAASAKKDGLPNVK
FR775239.1_[132829_133354]_-_       -----MKIRVFMATVLLLISHCVFS-----------------TTSLPHIV
CP009351.1_[884050_884524]_+_       ---------------------------------------------MKKIL
FP929062.1_[1670842_1671370]_+      ---------------------------------------------MKHLL
CP000407.1_[334649_335075]_-_       ---------------------------------------------MKKIL
CP003778.1_[4072337_4072823]_+      ---------------------------------------------MEDLL
AE008923.1_[1096145_1096631]_-      ---------------------------------------------MEDLL
CP004399.1_[1100060_1100546]_-      ---------------------------------------------MEDLL
CP006857.1_[1096139_1096625]_-      ---------------------------------------------MEDLL
CP008989.1_[1096143_1096629]_-      ---------------------------------------------MEDLL
CP008992.1_[1096143_1096629]_-      ---------------------------------------------MEDLL
CP008995.1_[1096145_1096631]_-      ---------------------------------------------MEDLL
CP008998.1_[1096147_1096633]_-      ---------------------------------------------MEDLL
CP009004.1_[1096155_1096641]_-      ---------------------------------------------MEDLL
CP009001.1_[1096150_1096636]_-      ---------------------------------------------MEDLL
CP009007.1_[1096136_1096622]_-      ---------------------------------------------MEDLL
CP009010.1_[1096138_1096624]_-      ---------------------------------------------MEDLL
CP009016.1_[1096154_1096640]_-      ---------------------------------------------MEDLL
CP009013.1_[1096126_1096612]_-      ---------------------------------------------MEDLL
CP009019.1_[1096155_1096641]_-      ---------------------------------------------MEDLL
CP009022.1_[1096145_1096631]_-      ---------------------------------------------MEDLL
CP009028.1_[1096145_1096631]_-      ---------------------------------------------MEDLL
CP009025.1_[1096140_1096626]_-      ---------------------------------------------MEDLL
CP009031.1_[4072159_4072645]_+      ---------------------------------------------MEDLL
CP009034.1_[4072160_4072646]_+      ---------------------------------------------MEDLL
CP009037.1_[4072154_4072640]_+      ---------------------------------------------MEDLL
CP009040.1_[4072108_4072594]_+      ---------------------------------------------MEDLL
AM039952.1_[1094639_1095125]_-      ---------------------------------------------MEDLL
CP002914.1_[1035664_1036150]_-      ---------------------------------------------MEDLL
CP003057.1_[982067_982553]_-_       ---------------------------------------------MEDLL
CP007221.1_[4092673_4093159]_+      ---------------------------------------------MEDLL
FO681494.1_[1080795_1081281]_-      ---------------------------------------------MEDLL
AP008229.1_[3882331_3882817]_+      ---------------------------------------------MEDLL
CP007166.1_[1135333_1135819]_-      ---------------------------------------------MEDLL
CP011256.1_[5126005_5126491]_-      ---------------------------------------------MEDLL
AE008922.1_[1016302_1016788]_-      ---------------------------------------------MEDLL
CP000050.1_[4023752_4024238]_+      ---------------------------------------------MEDLL
CP010409.1_[2013868_2014354]_+      ---------------------------------------------MEELL
FP565176.1_[530509_530995]_-_       ---------------------------------------------MEQLL
CP002986.1_[819984_820479]_-_       ------------------------------------------MHMMEELL
HE798556.1_[847871_848366]_-_       ------------------------------------------MHMMEELL
AM743169.1_[902805_903291]_-_       ---------------------------------------------MEELL
CP001111.1_[830691_831186]_-_       ------------------------------------------MHMMEELL
CP002446.1_[2592481_2592967]_+      ---------------------------------------------MEELL
CP011144.1_[894375_894861]_+_       ---------------------------------------------MEELL
AE009442.1_[474685_475171]_-_       ---------------------------------------------MEDLL
CP001011.1_[474538_475024]_-_       ---------------------------------------------MEDLL
CP002165.1_[1594542_1595028]_-      ---------------------------------------------MEDLL
AE003849.1_[1047304_1047790]_-      ---------------------------------------------MEDLL
CP000941.1_[485900_486386]_-_       ---------------------------------------------MEDLL
CP006740.1_[275355_275841]_-_       ---------------------------------------------MEDLL
CP003093.2_[2619491_2619977]_+      ---------------------------------------------MEDLL
CP003470.1_[1046951_1047443]_-      ---------------------------------------------MQHLT
CP008884.1_[4334510_4335002]_+      ---------------------------------------------MQHLT
CP007444.1_[3166036_3166528]_+      ---------------------------------------------MQHLT
CP003350.1_[866528_867014]_-_       ---------------------------------------------MQQLA
AP010904.1_[661932_662448]_+_       ------------------------------------------MGKHVKLA
CP003746.2_[1122651_1123134]_+      ----------------------------------------------MSVT
CP003985.1_[747499_748000]_-_       ----------------------------------------------MKII
CP001807.1_[750839_751322]_+_       ----------------------------------------------MDVK
CP003029.1_[2540593_2541118]_-      --------------------------------MFQIPARAADGSKNMDVK
CP002361.1_[27336_27843]_+_         --------------------------------MNPTPE-------PEFVQ
CP000356.1_[475998_476481]_+_       -----------------------------------------------MID
CP009122.1_[1397954_1398446]_+      --------------------------------------------MTDMID
CP000514.1_[3980723_3981203]_-      -----------------------------------------------MIE
FO203363.1_[3607326_3607806]_-      -----------------------------------------------MIE
CP001978.1_[3522100_3522580]_-      -----------------------------------------------MIQ
CP007151.1_[1684828_1685308]_+      -----------------------------------------------MIQ
AP010803.1_[2584265_2584769]_-      -----------------------------------------MPSPETPIL
CP002798.1_[1485579_1486083]_-      -----------------------------------------MLSPETPIL
AP012222.1_[2800176_2800680]_-      -----------------------------------------MTKSRDPIL
CP009291.1_[3253827_3254319]_-      -------------------------------------------MDRSPIL
FR856862.1_[1855341_1855833]_-      -------------------------------------------MDRSPIL
CP000248.1_[599766_600258]_+_       -------------------------------------------MQHQPIL
CP001087.1_[2132169_2132652]_-      ----------------------------------------------MKIK
FO203503.1_[3427664_3428150]_+      ----------------------------------------------MEIK
CP000230.1_[4281964_4282483]_+      -----------------------------------------MAVSPSPLR
CP003046.1_[4281964_4282483]_+      -----------------------------------------MAVSPSPLR
CP001131.1_[1967759_1968263]_+      -----------------------------------------MLPVSQPIR
CP001359.1_[2029760_2030264]_+      -----------------------------------------MRPVSQPIR
CP000251.1_[2419660_2420164]_-      -----------------------------------------MRPVSQPIR
CP000769.1_[4112838_4113333]_+      -------------------------------------------MATEPIR
AP009153.1_[2726509_2727019]_-      ----------------------------------------------MSLR
CP002691.1_[6510574_6511072]_-      -----------------------------------------------MVQ
CP002831.1_[2952159_2952681]_-      ------------------------------------------MKKNKSIL
CP003178.1_[7878322_7878829]_-      ----------------------------------------------MAIR
CP007035.1_[3690700_3691195]_-      ----------------------------------------------MAIR
CP000473.1_[8450546_8451041]_+      ---------------------------------------------MEPIR
CP001734.1_[541069_541603]_+_       ----------------------------------------MAQHPEKPIR
CP003221.1_[3710374_3710884]_+      ----------------------------------------MTKVADGRIR
CP002514.1_[2380426_2380936]_+      -----------------------------------------MSPTSPPLR
CP000269.1_[4096942_4097428]_+      ----------------------------------------------MTLR
CU207211.1_[3414611_3415097]_+      ----------------------------------------------MTLR
CP002745.1_[5163657_5164143]_+      ----------------------------------------------MALR
CP009962.1_[5575893_5576379]_+      ----------------------------------------------MALR
HG322949.1_[136787_137273]_-_       ----------------------------------------------MTLR
CP002039.1_[4937972_4938458]_+      ----------------------------------------------MSLR
CP002868.1_[1048763_1049252]_+      -------------------------MDG--------------------IR
CP003282.1_[779163_779724]_+_       -------------------------MNQPPDTRETLWRSPWSDSDDPELR
CP002541.1_[562683_563187]_-_       --------------------------------------------MAQELR
CP003155.1_[1580411_1580912]_+      --------------------------------------------MAQELR
CP002659.1_[299546_300053]_+_       -------------------------------------------MKDSRMR
CP006939.1_[1834369_1834888]_+      -------------------------------------------MKPVEIR
AP009247.1_[226235_226721]_+_       -------------------------------------------MR---IK
CP001100.1_[295450_295954]_+_       -----------------------------------------METK---IK
CP002776.1_[888646_889153]_-_       -----------------------------------------MAQ----IS
CP007030.1_[1079366_1079873]_+      -----------------------------------------MDA----IS
CP000109.2_[1350751_1351261]_+      -----------------------------------------MKASPLPIT
CP002959.1_[998274_998760]_-_       -----------------------------------------------MIR
CP004393.1_[4207116_4207608]_-      ---------------------------------------------MKDVR
CP001650.1_[1932398_1932884]_-      -----------------------------------------------MIQ
CU207366.1_[1270492_1270984]_+      -----------------------------------------------MIH
AP011540.1_[190666_191188]_+_       ----------------------------------MRKPVSVTIKNPEEIT
CP010848.1_[381590_382076]_-_       --------------------------------------------MTDRIL
AP009178.1_[1477696_1478179]_-      ------------------------------------------------MI
CP002452.1_[1342864_1343380]_+      --------------------------------------MPRLSNPLEGIA
CP000153.1_[583322_583808]_+_       ------------------------------------------------ML
CP002205.1_[1564141_1564627]_-      ------------------------------------------------ML
CP002355.1_[2175798_2176278]_-      ------------------------------------------------ML
CP003920.1_[2027875_2028355]_-      ------------------------------------------------ML
CP000361.1_[1593264_1593804]_-      -------------------------------MNFYNQKIWCKTMIKSKIT
CP006615.1_[1549239_1549779]_-      -------------------------------MNFYNQKIWCKTMIKSKIT
AP012047.1_[1512171_1512675]_-      -------------------------------------------MIKSKIT
AP012048.1_[2108336_2108825]_-      ----------------------------------------------MKIT
CP001999.1_[1168204_1168696]_+      ----------------------------------------------MTVT
CP003333.1_[452133_452628]_-_       ---------------------------------------------MEKIL
CP007201.1_[577476_577971]_-_       ---------------------------------------------MEKIL
CP001816.1_[424310_424805]_-_       ---------------------------------------------MEPIL
AP009179.1_[734918_735413]_-_       ---------------------------------------------MQKIL
CP001801.1_[2047872_2048406]_+      -------------------------------------MSTSNSPSSFPVV
CP002930.1_[3753879_3754398]_+      ---------------------------------MGTAIQ--------DVI
FQ312005.1_[1251955_1252498]_+      ---------------------------------MTADIENDVLNIAQDVI
CP002116.1_[274963_275503]_-_       ----------------------------------------------MKIT
CP002467.1_[871730_872243]_-_       ---------------------------------------------MRPIT
CP003379.1_[4474444_4474963]_+      ---------------------------------------------MHRVH
CP000394.1_[466380_466920]_-_       -------------------------------------MEPVMRSGPLSVL
CP003181.1_[468418_468958]_-_       -------------------------------------MEPVMRSGPLSVL
CP003182.1_[471874_472414]_-_       -------------------------------------MEPVMRSGPLSVL
CP003183.1_[466403_466943]_-_       -------------------------------------MEPVMRSGPLSVL
AP011115.1_[2298178_2298610]_-      --------------------------------------------------
CP000431.1_[2563714_2564146]_-      --------------------------------------------------
```

```
CP006890.1_[255482_256010]_+_      ILAAGGTIAGAAPSKTSATEYTAGA-IGVDALIQAVPEIKEV-AHVSGEQ
FR775239.1_[132829_133354]_-_      ILATGGTIAGTAANNTQTAGYKSGE-LGVQTLINAVPEMNNI-ARVDGEQ
CP009351.1_[884050_884524]_+_      LLATGGTIASVEGNEGLVPGLSA------EELLNYFSKSSRN-LEIDCKI
FP929062.1_[1670842_1671370]_+     LLGTGGTIACKRGENGLTPLLTG------DELLSYVPDAKKF-CEVETVQ
CP000407.1_[334649_335075]_-_      VLHTGGTISMQTNHQGEVAANKT-------NPMTQIDSPLEE-TQVTSLD
CP003778.1_[4072337_4072823]_+     IVTTGGTIDKIYFDDKSDYQIGDP---QIGQILKELGVTFR----FSVIP
AE008923.1_[1096145_1096631]_-     IVTTGGTIDKIYFDDKSDYQIGDP---QIGQILKELGVTFR----FSVIP
CP004399.1_[1100060_1100546]_-     IVTTGGTIDKIYFDDKSDYQIGDP---QIGQILKELGVTFR----FSVIP
CP006857.1_[1096139_1096625]_-     IVTTGGTIDKIYFDDKSDYQIGDP---QIGQILKELGVTFR----FSVIP
CP008989.1_[1096143_1096629]_-     IVTTGGTIDKIYFDDKSDYQIGDP---QIGQILKELGVTFR----FSVIP
CP008992.1_[1096143_1096629]_-     IVTTGGTIDKIYFDDKSDYQIGDP---QIGQILKELGVTFR----FSVIP
CP008995.1_[1096145_1096631]_-     IVTTGGTIDKIYFDDKSDYQIGDP---QIGQILKELGVTFR----FSVIP
CP008998.1_[1096147_1096633]_-     IVTTGGTIDKIYFDDKSDYQIGDP---QIGQILKELGVTFR----FSVIP
CP009004.1_[1096155_1096641]_-     IVTTGGTIDKIYFDDKSDYQIGDP---QIGQILKELGVTFR----FSVIP
CP009001.1_[1096150_1096636]_-     IVTTGGTIDKIYFDDKSDYQIGDP---QIGQILKELGVTFR----FSVIP
CP009007.1_[1096136_1096622]_-     IVTTGGTIDKIYFDDKSDYQIGDP---QIGQILKELGVTFR----FSVIP
CP009010.1_[1096138_1096624]_-     IVTTGGTIDKIYFDDKSDYQIGDP---QIGQILKELGVTFR----FSVIP
CP009016.1_[1096154_1096640]_-     IVTTGGTIDKIYFDDKSDYQIGDP---QIGQILKELGVTFR----FSVIP
CP009013.1_[1096126_1096612]_-     IVTTGGTIDKIYFDDKSDYQIGDP---QIGQILKELGVTFR----FSVIP
CP009019.1_[1096155_1096641]_-     IVTTGGTIDKIYFDDKSDYQIGDP---QIGQILKELGVTFR----FSVIP
CP009022.1_[1096145_1096631]_-     IVTTGGTIDKIYFDDKSDYQIGDP---QIGQILKELGVTFR----FSVIP
CP009028.1_[1096145_1096631]_-     IVTTGGTIDKIYFDDKSDYQIGDP---QIGQILKELGVTFR----FSVIP
CP009025.1_[1096140_1096626]_-     IVTTGGTIDKIYFDDKSDYQIGDP---QIGQILKELGVTFR----FSVIP
CP009031.1_[4072159_4072645]_+     IVTTGGTIDKIYFDDKSDYQIGDP---QIGQILKELGVTFR----FSVIP
CP009034.1_[4072160_4072646]_+     IVTTGGTIDKIYFDDKSDYQIGDP---QIGQILKELGVTFR----FSVIP
CP009037.1_[4072154_4072640]_+     IVTTGGTIDKIYFDDKSDYQIGDP---QIGQILKELGVTFR----FSVIP
CP009040.1_[4072108_4072594]_+     IVTTGGTIDKIYFDDKSDYQIGDP---QIGQILKELGVTFR----FSVIP
AM039952.1_[1094639_1095125]_-     IVTTGGTIDKIYFDDKSDYQIGDP---QIGQILKELGVTFR----FSVIP
CP002914.1_[1035664_1036150]_-     IVTTGGTIDKIYFDDKSDYQIGDP---QIGQILKELGVTFR----FSVIP
CP003057.1_[982067_982553]_-_      IVTTGGTIDKIYFDDKSDYQIGDP---QIGQILRELGVTFR----FSVIP
CP007221.1_[4092673_4093159]_+     IVTTGGTIDKIYFDDKSDYQIGDP---QIGQILRELGVTFR----FSVIP
FO681494.1_[1080795_1081281]_-     IVTTGGTIDKIYFDDKSDYQIGDP---QIGQILKELGVTFR----FSVIP
AP008229.1_[3882331_3882817]_+     IVTTGGTIDKIYFDDKSDYQIGDP---QIGQILKELGVTFR----FSVTP
CP007166.1_[1135333_1135819]_-     IVTTGGTIDKIYFDDKSDYQIGDP---QIGQILKELGVTFR----FSVTP
CP011256.1_[5126005_5126491]_-     IVTTGGTIDKIYFDDKSDYQIGDP---QIGQILKELGVTFR----FSVIP
AE008922.1_[1016302_1016788]_-     IVTTGGTIDKIYFDDKSDYQIGDP---QIGQILKELGVTFR----FTVIP
CP000050.1_[4023752_4024238]_+     IVTTGGTIDKIYFDDKSDYQIGDP---QIGQILKELGVTFR----FTVIP
CP010409.1_[2013868_2014354]_+     IVTTGGTIDKIYFDDKSDYQIGDP---QIGQILKELGVTFR----FTVIP
FP565176.1_[530509_530995]_-_      IVTTGGTIDKIYFDDKSDYQIGDP---QIGQILKELGVTFR----FSVIP
CP002986.1_[819984_820479]_-_      VVTTGGTIDKIYFDDKSDYQIGDP---QIGMILRELGVTFR----FNVIP
HE798556.1_[847871_848366]_-_      VVTTGGTIDKIYFDDKSDYQIGDP---QIGMILRELGVTFR----FNVIP
AM743169.1_[902805_903291]_-_      VVTTGGTIDKIYFDDKSDYQIGDP---QIGMILRELGVTFR----FNVIP
CP001111.1_[830691_831186]_-_      VVTTGGTIDKIYFDDKSDYQIGDP---QIGMILRELGVTFR----FNVIP
CP002446.1_[2592481_2592967]_+     IVTTGGTIDKIYFDDKSDYQIGEP---QIGMILRELGVNFR----FSVIP
CP011144.1_[894375_894861]_+_      IVTTGGTIDKIYFDDKSDYQIGEP---QIGTILRELGVSFR----FSVIP
AE009442.1_[474685_475171]_-_      IVTTGGTIDKIYFDEKSHYQVGDP---QITQILEALGIAFR----FNVIP
CP001011.1_[474538_475024]_-_      IVTTGGTIDKIYFDEKSHYQVGDP---QITQILEALGIAFR----FNVIP
CP002165.1_[1594542_1595028]_-     IVTTGGTIDKIYFDEKSHYQVGDP---QITQILEALGIAFR----FNVIP
AE003849.1_[1047304_1047790]_-     IVTTGGTIDKIYFDEKSHYQVGDP---QITQILEALGIAFR----FNVIP
CP000941.1_[485900_486386]_-_      IVTTGGTIDKIYFDEKSHYQIGDP---QITQILEALGIAFR----FNVIP
CP006740.1_[275355_275841]_-_      IVTTGGTIDKIYFDEKSHYQIGDP---QITQILEALGIAFR----FNVIP
CP003093.2_[2619491_2619977]_+     VVTTGGTIDKIYFDDKSDYQVGEP---QIGGILKALGVAFG----FTVIP
CP003470.1_[1046951_1047443]_-     IVTTGGTIDKIYFDDKSDYKIGAP---QIGEILGQLGVAFQ----FDVIP
CP008884.1_[4334510_4335002]_+     IVTTGGTIDKIYFDDKSDYKIGSP---QIGDILSQLGVAFQ----FDVIP
CP007444.1_[3166036_3166528]_+     IVTTGGTIDKIYFDDKSDYKIGAP---QIGEILGQLGVAFQ----FDVIP
CP003350.1_[866528_867014]_-_      IITTGGTIDKIYFDDKSDYKIGAP---QISDILGQFGVAFD----YEVHA
AP010904.1_[661932_662448]_+_      VYSMGGTIDKVYFDDLSDYSVGDP---QVGAILTQAGVDFT----WSVTE
CP003746.2_[1122651_1123134]_+     IITTGGTIDKIYYDAKNDYHIGQP---QAPKILERAGVTLD----TPLIP
CP003985.1_[747499_748000]_-_      IYTTGGTIDKVYFDQKSEYQVGDP---QANGVLERANVVLD----YVVES
CP001807.1_[750839_751322]_+_      IFTTGGTIDKVYFDAKSTYEVGDP---QIVDILREANVTID----WELET
CP003029.1_[2540593_2541118]_-     IFTTGGTIDKVYFDAKSTYEVGDP---QIVDILREANVTID----WELET
CP002361.1_[27336_27843]_+_        VFTTGGTIDKVYYDAKDDYTVGDP---QIGKLLEEARVNFP----YAIEA
CP000356.1_[475998_476481]_+_      IFTTGGTIDKVYFDALSEFQIGPA---AIPDMLRENNVLVP----HRVTQ
CP009122.1_[1397954_1398446]_+     IFTTGGTIDKVYFDALSEFQIGPA---AIPDMLRENNVHVP----HRVTQ
CP000514.1_[3980723_3981203]_-     ILTTGGTIDKVYFDANSKFEIGDS---LLPELLTESNIHDG----YEIRP
FO203363.1_[3607326_3607806]_-     ILTTGGTIDKVYFDANSKFEIGDS---LLPELLTESNIHDG----YEIRP
CP001978.1_[3522100_3522580]_-     IFTTGGTIDKVYFDANSEFEVGHS---LLPELLSESNIHEG----YRLRE
CP007151.1_[1684828_1685308]_+     IFTTGGTIDKVYFDANSQFEVGTS---LLPELLAESNIHDG----YRVQE
AP010803.1_[2584265_2584769]_-     VLTTGGTIDKIYFDALSDYQVGET---VVEKLLDIGRVKRP----FRIEE
CP002798.1_[1485579_1486083]_-     VLTTGGTIDKIYFDALSDYQVGET---VMAKLLDIARVKRP----FRIEE
AP012222.1_[2800176_2800680]_-     VLTTGGTIDKLYFDALSEYRIGES---ILARVLDAARVTHP----VEIVE
CP009291.1_[3253827_3254319]_-     VVTTGGTIDKQYFDALSEYQITES---VIEKLLKIARVTHP----FRIVE
FR856862.1_[1855341_1855833]_-     VVTTGGTIDKQYFDALSEYQITES---VIEKLLKIARVTHP----FRIVE
CP000248.1_[599766_600258]_+_      VLTTGGTIDKAYFDALSEYQIVES---GIPALIAEARVALP----FRIVE
CP001087.1_[2132169_2132652]_-     FFTLGGTIDKVYFDAMSRYEVGPP---RVKEILKEARVNFE----YSIKS
FO203503.1_[3427664_3428150]_+     FIAVGGTIDKVYFDSLSRYEIGES---NINDILKDGRINFK----YKVSS
CP000230.1_[4281964_4282483]_+     IFTAGGTIDKDYRLEENGLVVGDP---FVAEVLKTARLAGA----VSIVA
CP003046.1_[4281964_4282483]_+     IFTAGGTIDKDYRLEENGLVVGDP---FVAEVLKTARLAGA----VSIVA
CP001131.1_[1967759_1968263]_+     VFVTGGTFDKEYDELSGRLYFKDT---HLPEMLRLARCRLD----VSVRN
CP001359.1_[2029760_2030264]_+     VFVTGGTFDKEYDELSGRLYFKDT---HLPEMLRLARCRLD----VSVRN
CP000251.1_[2419660_2420164]_-     VFVTGGTFDKEYDELSGRLYFKDT---HLPEMLRLARCRLE----VSVRN
CP000769.1_[4112838_4113333]_+     LFVTGGTFDKEYDELSGRLYFKDS---HVHEMLRLARCRVE----TRVQT
AP009153.1_[2726509_2727019]_-     VFVTGGTFDKEYDELTGTLRFRET---HLPEMLRRGRSMLD----VQVEV
CP002691.1_[6510574_6511072]_-     IFVTGGTFDKEYNYIEGKLYFQDT---HLPKMLVTGRCTLD----IDVKT
CP002831.1_[2952159_2952681]_-     ALVTGGTFDKEYDMITGKLFFKDT---HLSEIFKRGRATLD----IKVRT
CP003178.1_[7878322_7878829]_-     IFITGGTFDKEYNELNGELYFKDT---HMDELLAEGRSRLN----VHIKT
CP007035.1_[3690700_3691195]_-     IFITGGTFDKEYNMLNGELYFKDT---HLQELLEKGRNTVP----VEIRT
CP000473.1_[8450546_8451041]_+     ILVTGGTFDKEYNELTGELFFQKT---HAGDMLKLGRCLLE----VEIET
CP001734.1_[541069_541603]_+_      ILITGGTIDKDYNKLAGQLVLGKT---HLQDILEQARCRVP----VRVEQ
CP003221.1_[3710374_3710884]_+     FLITGGTIDKEYDAQSGNLVFSRT---RLGDILTLARCRAE----FVVEV
CP002514.1_[2380426_2380936]_+     ILLTGGTLDKVYDERTGNLVFSRT---HLFDMLDQARCYLAN---ITIEP
CP000269.1_[4096942_4097428]_+     IIATGGTFDKHYDEITGQLNFSHG---HLPQVIKRARITVP----LQLEQ
CU207211.1_[3414611_3415097]_+     IIATGGTFDKHYDEIAGKLNFSKG---HLPQIIKRCRITVP----IQLEQ
CP002745.1_[5163657_5164143]_+     IIATGGTFDKHYDEIAGKLTFATS---HLPQVIQRARITTE----IALEE
CP009962.1_[5575893_5576379]_+     IIATGGTFDKHYDEIAGKLTFAAS---HLPQVIQRARITAE----IALEE
HG322949.1_[136787_137273]_-_      ILATGGTFDKHYNELNGTLGFSDS---HLPAAIARARMTAP----VALEQ
CP002039.1_[4937972_4938458]_+     IIATGGTFDKHYDEIAGQLGFGES---HLPDVIARARITAE----VSLEI
CP002868.1_[1048763_1049252]_+     ILITGGTFDKHYDELKGVLTFKNS---HLPDIIKQVRITVP----VELEI
CP003282.1_[779163_779724]_+_      IIITGGTFDKRYDAISGQLGFAGT---HLPDILQRVRCSYP----VVLEL
CP002541.1_[562683_563187]_-_      IITTGGTFDKQYDAISGELTFRES---QLPRILGQSRCTLT----VHLEG
CP003155.1_[1580411_1580912]_+     IITTGGTFDKQYDAIKGELTFRES---QLPRILQQARCTLT----VQLEG
CP002659.1_[299546_300053]_+_      IITTGGTFDKQYDAITGELTFRSS---QLPRLLLEARCTLE----THLVG
CP006939.1_[1834369_1834888]_+     IIITGGTFDKEYDPLKGELSFRDS---HLPGILKTVRCTLE----TSLEI
AP009247.1_[226235_226721]_+_      LLITGGTIDKVYDELIGQLVFQRT---YLIDMLNRSRSMVD----TLSEV
CP001100.1_[295450_295954]_+_      LFITGGTIDKKYNELDGSLGFHQT---HFPAMLSQARNAAS----VTLDI
CP002776.1_[888646_889153]_-_      ILACGGTLDKDYDPLTGELVFGHT---HLMELLNQANHTLH----ISVQE
CP007030.1_[1079366_1079873]_+     ILACGGTLDKDYDPLRGELVFSQS---HLPTLLAQANHTLP----LSVNA
CP000109.2_[1350751_1351261]_+     LLITGGTLDKDYQATTGDLIFSET---HLPKLLKEANSTLP----IKLKV
CP002959.1_[998274_998760]_-_      ILATGGTLDVDSIDEGDVYYFQES---YVPKILAQCRNEAD----IVVET
CP004393.1_[4207116_4207608]_-     IIVTGGTIDKVHDMQGEGLGFAPSGASHIPLLLEQARCFFP-----KVQE
CP001650.1_[1932398_1932884]_-     IFTTGGTIEGLDYVDGKGIT-QSSVT--IETFLEKANVDFE----YAVES
CU207366.1_[1270492_1270984]_+     IITTGGTIEGLDYENPENKTEQSEIA--IREFLDLANVSFP----YIIER
AP011540.1_[190666_191188]_+_      ILATGGTIDKFYS-VAGTMDIGEP---AAKDLLDRVITDIR----FDIRP
CP010848.1_[381590_382076]_-_      LLTTGGTIDKEYS-LAGELEIGEP---TVSRLLEPVLTTLE----FRSEQ
AP009178.1_[1477696_1478179]_-     ILNTGGTFNKIYDPIKGELVVPQN-----NDAIYDFLKKNF--LQIETRG
CP002452.1_[1342864_1343380]_+     ILSTGGTFNKIYDPIAGELRVDPH-----SQAVQELLKHWK--CQAPLIN
CP000153.1_[583322_583808]_+_      ILNSGGTFNKKYNSLNGELEIPYN-----NSVIEEILKSVE--FKYDLAG
CP002205.1_[1564141_1564627]_-     ILNSGGTFNKRYNEISGELEVPYD-----NAAIERILYSTQ--EIYNLAG
CP002355.1_[2175798_2176278]_-     IINTGGTFNKRYDPIKGELFVPND-----NQAVESILESLV--IDISLQG
CP003920.1_[2027875_2028355]_-     ILNTGGTFNKRYDSLKGELFVPND-----NRAVEAVLKSLV--IDIPLQG
CP000361.1_[1593264_1593804]_-     VLNTGGTFNKRYNPLKGELEVPSD-----NIALDEIIQSCFN-IDFEIIN
CP006615.1_[1549239_1549779]_-     VLNTGGTFNKRYNPLKGELEVPTD-----NIALDEIIQSCFN-VDFEIIN
AP012047.1_[1512171_1512675]_-     VLNTGGTFNKRYNPLKGELEVPTD-----NIALDEIIQNCFN-VDFEIIN
AP012048.1_[2108336_2108825]_-     LINTGGTFNKRYNPLKGELEVPKD-----NIALDEIIESCFN-IEFEIKN
CP001999.1_[1168204_1168696]_+     ILNTGGTFNKRYNPIKGELEVPTD-----NLALDKIIKSCHN-VTFDIKN
CP003333.1_[452133_452628]_-_      IMNTGGTFNKRYNPLKGELEVPKD-----GMAVESILRYCPN-MQYTLCN
CP007201.1_[577476_577971]_-_      ILNTGGTFNKRYNPLKGELEVPQD-----GIAVESILRYCYN-TSYELLN
CP001816.1_[424310_424805]_-_      ILNTGGTFNKRYNPLRGELEVPKD-----SNAIESILHHCSN-THYTLRS
AP009179.1_[734918_735413]_-_      IISTGGTFNKIYDPIVGDLIVEKS-----SKAVKEIASKWL--SRFEVLS
CP001801.1_[2047872_2048406]_+     IINTGGTFNKRYQPLTGLLTVAND-----ERTIEELLHSASPNLDMHLIG
CP002930.1_[3753879_3754398]_+     IITTGGTIEKTYNEFDGSLENRGT--SIKNRILSKMRLPYTN---IMVYP
FQ312005.1_[1251955_1252498]_+     VLTTGGTIEKSYDEYDGSLENKDS--IIKHIILQKLRMPYTK---LHVHS
CP002116.1_[274963_275503]_-_      ILTTGGTIDKTYDEHAGSLRN-ER--TVLDTILKSLRLPDLF---IRHVA
CP002467.1_[871730_872243]_-_      LLTTGGTIEKRYVEQMGAMANTEP---QIQRCLALLRLPGAK---ITVEE
CP003379.1_[4474444_4474963]_+     LITTGGTIEKRYVEQDGSMENTVP---QIRRCLSMLRLPDTA---ITVEE
CP000394.1_[466380_466920]_-_      IVSTGGTISKRYVEATGALDPHPD---WLAALGPRLRLPQT---EITMVQ
CP003181.1_[468418_468958]_-_      IVSTGGTISKRYVEATGALDPHPD---WLAALGPRLRLPQT---EITMVQ
CP003182.1_[471874_472414]_-_      IVSTGGTISKRYVEATGALDPHPD---WLAALGPRLRLPQT---EITMVQ
CP003183.1_[466403_466943]_-_      IVSTGGTISKRYVEATGALDPHPD---WLAALGPRLRLPQT---EITMVQ
AP011115.1_[2298178_2298610]_-     MITNVSLVTLWVTDQDDAKKFYID-----------------------TLG
CP000431.1_[2563714_2564146]_-     MITNVSLVTLWVTDQDDAKKFYID-----------------------TLG
                                          .  .
```

```
CP006890.1_[255482_256010]_+_       VVNIGSQNMNNETLLKLAKRVNKLLKSDDTDGIVITHGTDTLEETAYFLN
FR775239.1_[132829_133354]_-_       VANIGSENMTSDIILKLSQKVNALLARDDVDGVVITHGTDTLDETAYFLR
CP009351.1_[884050_884524]_+_       LMNIDSTNMQPEHWKEIANAVFNHY--DDYDGFVITHGTDTLAYTSSALS
FP929062.1_[1670842_1671370]_+      VLNIDSTNMHPKHWLKLAQVLEENY--DDYDGFVICHGTDTMAYTAAAMS
CP000407.1_[334649_335075]_-_       FLNVPSPHIRLEHMMTLYQKIKEEQ--MNFDGFVITHGTDTLEETAYFLD
CP003778.1_[4072337_4072823]_+      IIRKDSLHITDADRELIRATIAAQP----TRHVLITHGTDSMVETGKVLQ
AE008923.1_[1096145_1096631]_-      IIRKDSLHITDADRELIRATIAAQP----TRHVLITHGTDSMVETGKVLQ
CP004399.1_[1100060_1100546]_-      IIRKDSLHITDADRELIRATIAAQP----TRHVLITHGTDSMVETGKVLQ
CP006857.1_[1096139_1096625]_-      IIRKDSLHITDADRELIRATIAAQP----TRHVLITHGTDSMVETGKVLQ
CP008989.1_[1096143_1096629]_-      IIRKDSLHITDADRELIRATIAAQP----TRHVLITHGTDSMVETGKVLQ
CP008992.1_[1096143_1096629]_-      IIRKDSLHITDADRELIRATIAAQP----TRHVLITHGTDSMVETGKVLQ
CP008995.1_[1096145_1096631]_-      IIRKDSLHITDADRELIRATIAAQP----TRHVLITHGTDSMVETGKVLQ
CP008998.1_[1096147_1096633]_-      IIRKDSLHITDADRELIRATIAAQP----TRHVLITHGTDSMVETGKVLQ
CP009004.1_[1096155_1096641]_-      IIRKDSLHITDADRELIRATIAAQP----TRHVLITHGTDSMVETGKVLQ
CP009001.1_[1096150_1096636]_-      IIRKDSLHITDADRELIRATIAAQP----TRHVLITHGTDSMVETGKVLQ
CP009007.1_[1096136_1096622]_-      IIRKDSLHITDADRELIRATIAAQP----TRHVLITHGTDSMVETGKVLQ
CP009010.1_[1096138_1096624]_-      IIRKDSLHITDADRELIRATIAAQP----TRHVLITHGTDSMVETGKVLQ
CP009016.1_[1096154_1096640]_-      IIRKDSLHITDADRELIRATIAAQP----TRHVLITHGTDSMVETGKVLQ
CP009013.1_[1096126_1096612]_-      IIRKDSLHITDADRELIRATIAAQP----TRHVLITHGTDSMVETGKVLQ
CP009019.1_[1096155_1096641]_-      IIRKDSLHITDADRELIRATIAAQP----TRHVLITHGTDSMVETGKVLQ
CP009022.1_[1096145_1096631]_-      IIRKDSLHITDADRELIRATIAAQP----TRHVLITHGTDSMVETGKVLQ
CP009028.1_[1096145_1096631]_-      IIRKDSLHITDADRELIRATIAAQP----TRHVLITHGTDSMVETGKVLQ
CP009025.1_[1096140_1096626]_-      IIRKDSLHITDADRELIRATIAAQP----TRHVLITHGTDSMVETGKVLQ
CP009031.1_[4072159_4072645]_+      IIRKDSLHITDADRELIRATIAAQP----TRHVLITHGTDSMVETGKVLQ
CP009034.1_[4072160_4072646]_+      IIRKDSLHITDADRELIRATIAAQP----TRHVLITHGTDSMVETGKVLQ
CP009037.1_[4072154_4072640]_+      IIRKDSLHITDADRELIRATIAAQP----TRHVLITHGTDSMVETGKVLQ
CP009040.1_[4072108_4072594]_+      IIRKDSLHITDADRELIRATIAAQP----TRHVLITHGTDSMVETGKVLQ
AM039952.1_[1094639_1095125]_-      IIRKDSLHITDADRELIRATIAAQP----TRHVLITHGTDSMVETGKVLQ
CP002914.1_[1035664_1036150]_-      IIRKDSLHITDADRELIRATIAAQP----TRHVLITHGTDSMVETGKVLQ
CP003057.1_[982067_982553]_-_       IIRKDSLHITDADRELIRATIAAQP----TCHVLITHGTDSMVETGKVLQ
CP007221.1_[4092673_4093159]_+      IIRKDSLHITDADRELIRATIAAQP----TCHVLITHGTDSMVETGKVLQ
FO681494.1_[1080795_1081281]_-      IIRKDSLHITDADRELIRATIAAQP----TRHVLITHGTDSMVETGKVLQ
AP008229.1_[3882331_3882817]_+      IIRKDSLHITDADRELIRATIAAQP----IRHVLITHGTDSMVETGKVLQ
CP007166.1_[1135333_1135819]_-      IIRKDSLHITDADRELIRATIAAQP----TRHVLITHGTDSMVETGKVLQ
CP011256.1_[5126005_5126491]_-      IIRKDSLHITDADRELVRATIAAQP----TRHVLITHGTDSMVETGKVLQ
AE008922.1_[1016302_1016788]_-      IIRKDSLHITQEDRELIRATIQAQP----ARQVLITHGTDSMVETGKVLQ
CP000050.1_[4023752_4024238]_+      IIRKDSLHITQEDRELIRATIQAQP----ARQVLITHGTDSMVETGKVLQ
CP010409.1_[2013868_2014354]_+      IIRKDSLHITDDDRELIRATIAAQS----ARHVLITHGTDSMVQTGKVLQ
FP565176.1_[530509_530995]_-_       ILRKDSLHITDADRELIRATVAAQS----ARHVLITHGTDSMVQTGKALQ
CP002986.1_[819984_820479]_-_       ILRKDSLHINDEDRELIRATIAAQP----TRHVLVTHGTDSMVQTGKVLA
HE798556.1_[847871_848366]_-_       ILRKDSLHINDEDRELIRATIAAQP----TRHVLVTHGTDSMVQTGKVLA
AM743169.1_[902805_903291]_-_       ILRKDSLHINDEDRELIRATIAAQP----TRHVLVTHGTDSMVQTGKVLA
CP001111.1_[830691_831186]_-_       ILRKDSLHINDEDRELIRATIAAQP----TRHVLVTHGTDSMVQTGQVLA
CP002446.1_[2592481_2592967]_+      ILRKDSLHITDADRELVRATIAAQP----ARHVLVTHGTDSMVQTGLVLK
CP011144.1_[894375_894861]_+_       ILRKDSLHITDEDRELVRATIAAQP----TRHVLVTHGTDSMVQTGLVLK
AE009442.1_[474685_475171]_-_       IMRKDSLHMVEADRELIHNTITAHS----CQHVLITHGTDSMVETGKRLQ
CP001011.1_[474538_475024]_-_       IMRKDSLHMVEADRELIHNTITAHS----CQHVLITHGTDSMVETGKRLQ
CP002165.1_[1594542_1595028]_-      IMRKDSLHMVEADRELIHNTITAHS----CQHVLITHGTDSMVETGKRLQ
AE003849.1_[1047304_1047790]_-      IMRKDSLHMVEADRELIHNTITAHP----CQHVLMTHGTDSMVETGKRLQ
CP000941.1_[485900_486386]_-_       IMRKDSLHMVEADRELIHNTITAHP----CQHVLITHGTDSMVETGKRLQ
CP006740.1_[275355_275841]_-_       IMRKDSLHMVEADRELIHNTITAHP----CQHVLITHGTDSMVETGKRLQ
CP003093.2_[2619491_2619977]_+      LIRKDSLHITDADRALLRATIAAQP----TRHVLITHGTDSMVQTAQALA
CP003470.1_[1046951_1047443]_-      ILRKDSLHMGAEDRALVRSTIEAQP----HRHVLVTHGTDTMVETARELA
CP008884.1_[4334510_4335002]_+      ILRKDSLHMGDEDRALVRSTIEAQP----HRHVLVTHGTDTMVETARELA
CP007444.1_[3166036_3166528]_+      ILRKDSLHITEEDRALVRSTIEAQP----HRHVLVTHGTDTMVETARELS
CP003350.1_[866528_867014]_-_       LMRKDSLHITDEDRAQILATVKAIP----QDRILITHGTDSMVETARVLQ
AP010904.1_[661932_662448]_+_       IARKDSLYVTDEERAVLRGRILADA----AGHVLITHGTDTMTRTAEFLA
CP003746.2_[1122651_1123134]_+      LFRKDSLDITDEDRALIRSTVEQQP----GHNIVITHGTDTMTKTGVFLQ
CP003985.1_[747499_748000]_-_       IMKKDSLDFTDEDRELIRRKVESSS----FERIVITHGTDTMIATAKVLK
CP001807.1_[750839_751322]_+_       LFRKDSLDLTDEDRAVIVEKVRQEP----CPRILITHGTDTMIETAKALQ
CP003029.1_[2540593_2541118]_-      LFRKDSLDLTDEDRAVIVEKVRRES----CPRILITHGTDTMIETAKALQ
CP002361.1_[27336_27843]_+_         LFKKDSLQMTDADRARIREAVAAAP----ARRVLITHGTDTMVETARALV
CP000356.1_[475998_476481]_+_       LMRKDSLELTDADREAIRAAVAASD----AARILVTHGTDTMVVTGRVLA
CP009122.1_[1397954_1398446]_+      LMRKDSLELDDADREAIRAAVAASD----ASRILVTHGTDTMVVTGRVLA
CP000514.1_[3980723_3981203]_-      LMRKDSLELTDEDRQAILEAVKASA----CQRILITHGTDTMAQTAEALK
FO203363.1_[3607326_3607806]_-      LMRKDSLELTDEDRQAILEAVKASA----CQRILITHGTDTMAQTAEALK
CP001978.1_[3522100_3522580]_-      LLRKDSLEMTDDDRQQVLAAATECD----CGRIIITHGTDTMADTAAVLS
CP007151.1_[1684828_1685308]_+      LMRKDSLEMTDADRQAVLEAVSASE----CDRILITHGTDTMPDTAAALA
AP010803.1_[2584265_2584769]_-      VVRKDSLELDDADRALIRDRVAAAP----ESRIIITHGTDTMTETAKSLS
CP002798.1_[1485579_1486083]_-      VTRKDSLELDDADRALIRDRVAAAP----ESHIIITHGTDTMTETAKLLS
AP012222.1_[2800176_2800680]_-      LLRKDSLDLDDADRALITERVAAAP----GRPVVITHGTDTMTQTAAELA
CP009291.1_[3253827_3254319]_-      LMRKDSLELTDEDRALIAATIANAP----EKRVVVTHGTDTMAVTAQALA
FR856862.1_[1855341_1855833]_-      LMRKDSLELTDEDRALIAATIANAP----EKRVVVTHGTDTMAVTAQALA
CP000248.1_[599766_600258]_+_       LMRKDSLELTDADRALIAAAVREAP----ETHIVITHGTDTMTDTAKALA
CP001087.1_[2132169_2132652]_-      LAKKDSLDMTDADRKRLVDALLTDD----CDRIVVTHGTDTMVDTAMALK
FO203503.1_[3427664_3428150]_+      ILKKDSLDMTDQDRLIIHQAVQAEI----NDKIIITHGTDTMIETAKVLA
CP000230.1_[4281964_4282483]_+      LSRKDSLDFTEADREAIGRAVGQAV----EDHILLTHGTDTMVETARYLG
CP003046.1_[4281964_4282483]_+      LSRKDSLDFTEADREAIGRAVGQAV----EDHILLTHGTDTMVETARYLG
CP001131.1_[1967759_1968263]_+      LMMVDSLDMTDGDRRIIVESCRSAA----EDRILVTHGTDTMAETARALL
CP001359.1_[2029760_2030264]_+      LMMVDSLDMTDGDRRIIVESCRSAA----EDRILVTHGTDTMAETARALL
CP000251.1_[2419660_2420164]_-      LMMVDSLDMTDGDRRIIVESCRAAA----EDRIVVTHGTDTMAETARALL
CP000769.1_[4112838_4113333]_+      LMMIDSLDMTEADRATVVESCRSAA----ERRIVVTHGTDTMPETARALL
AP009153.1_[2726509_2727019]_-      LMMIDSLEMLGSHRQQIAEACARAP----ESRLVITHGTDTMPETAQHLA
CP002691.1_[6510574_6511072]_-      LMMIDSLEMTDADRSIIAHNCQRNE----HARILITHGTDTIVQTATYLA
CP002831.1_[2952159_2952681]_-      LMMVDSLEMTDLDRDIIIKHCQSTE----EQQILITHGTDTMEVTAAAIA
CP003178.1_[7878322_7878829]_-      LMMIDSLEMTSDHRNLIAEECQQCD----EDRIVITHGTDTMAETARVLA
CP007035.1_[3690700_3691195]_-      LMMVDSLEMTDEDRELIAYQCEQSE----EDQIVITHGTDTMSVTAQLLS
CP000473.1_[8450546_8451041]_+      LMMIDSLQMTDADRLHILDRCAAVP----EKRLVITHGTDTMEQTAAVLG
CP001734.1_[541069_541603]_+_       LMLVDSLEMTEENRQTVLQRCVDAP----EERIIITHGTDTMVETARLLG
CP003221.1_[3710374_3710884]_+      VMLKDSLDMTMEDRLLIRERCLAAP----ESRIVITHGTDTMADTARVLG
CP002514.1_[2380426_2380936]_+      VMLKDSLDMTEADRAIIRAAVARAP----EHHIIVTHGTDTMTETAQVVA
CP000269.1_[4096942_4097428]_+      LPFLDSLDMHDADRQRIAASCLRAD----ETSIVVVHGTDTMRETAEVLG
CU207211.1_[3414611_3415097]_+      LPFLDSLDMHDADRQRIAASCLRAD----ETSIVVVHGTDTMRETAEVLG
CP002745.1_[5163657_5164143]_+      LPLLDSLDMQDIDRRRVLASCTHAA----EAAIVIIHGTDTMRETAAVLG
CP009962.1_[5575893_5576379]_+      LPLLDSLDMQDIDRRRVLASCTHAK----EEAIVIIHGTDTMRETAAVLG
HG322949.1_[136787_137273]_-_       LPLLDSLDMQDADRQRVLASCRAAS----EKAIVIIHGTDTMRETAQVLG
CP002039.1_[4937972_4938458]_+      LPLLDSLEMQDGDRQRVLAACQASS----QQAIVIVHGTDTMQQTAQVLG
CP002868.1_[1048763_1049252]_+      LQLVDSLEMGDQDRQNILAACRKTP----EDRIVITHGTDRMVDTAKVLG
CP003282.1_[779163_779724]_+_       NQLIDSLDMQPEHHRGIVDSCRRSP----QRRIVITHGTDRLQHTARELC
CP002541.1_[562683_563187]_-_       PLAVDSLYMTEEQRHEIARTCLHSA----EDRIIVVHGTDTMCNTAKVIA
CP003155.1_[1580411_1580912]_+      PLAIDSLYMTEEQRHEVAASCLSSN----EDRIVVIHGTDTMCKTAAVVA
CP002659.1_[299546_300053]_+_       PVAVDSLVMTEEQRNEIARECSQSP----ENMVVVIHGTDTMIATATIVA
CP006939.1_[1834369_1834888]_+      NELTDSLDMTDVHREGIAKSCLASP----QKRIVITHGTDTMTLTAEYLQ
AP009247.1_[226235_226721]_+_       LFLKDSLDMSDIDRALIVKKCQESD----TSHILITHGTDSMIRTAKLLA
CP001100.1_[295450_295954]_+_       ILMKDSLEMNDTDRALICQKCLECE----ETLILISHGTDTMVETGKFLS
CP002776.1_[888646_889153]_-_       IMLKDSLLMDDADRDLLYQACSKTP----NQQIVITHGTDTMPETATYLA
CP007030.1_[1079366_1079873]_+      VMLKDSLMMDDTDRESIYQACLADH----SEQIVITHGTDTMPETAIYIA
CP000109.2_[1350751_1351261]_+      LMLKDSLELLDSDRQKILDACLESE----TDRIVITHGTDTMTDTALFLK
CP002959.1_[998274_998760]_-_       LFLKDSLYLTDLDRQQILQHAKTCD----ELKIVVTHGTDTMVETATVLG
CP004393.1_[4207116_4207608]_-      ILLKDSLDFDEADRAAILFAVRNAP----ETAIVVTHGTGTIGRTARELA
CP001650.1_[1932398_1932884]_-      VFKKDSRAIDEKDIQLLVQRIRNAN----STKILITHGTFTMEDTAKYIG
CU207366.1_[1270492_1270984]_+      AFSKDSRSITDEDRNFLLKKIKASN----AEKILITHGTFTMEDTATYFG
AP011540.1_[190666_191188]_+_       LIGKDSLDMTDEDRAELTEALNAVT----NDQVLITHGTDTMPETARYLV
CP010848.1_[381590_382076]_-_       VLRKDSLDLDASDRALLKQRVLSAE----EDRIVITHGTDTMTETAEAIG
AP009178.1_[1477696_1478179]_-      LIYKDSLEFSDADRELLLQTIKNCD----SKQIVVVHGTDTMDKSAAFVA
CP002452.1_[1342864_1343380]_+      IIGKDSLEMNEEDRRLLAQTVQERP----ERKILIVHGTDTMDLSGAHLA
CP000153.1_[583322_583808]_+_       VVYKDSLDMNMDDRKRIAHIIMESS----DDTFIIVHGTDTMLLSAEFLS
CP002205.1_[1564141_1564627]_-      VVYKDSLDMDTNDRRMLADIIRESS----EDTFVIVHGTDTMDLSAEFLD
CP002355.1_[2175798_2176278]_-      VIYKDSLEMDAQDRTFLGQTIAASD----AQTVIVIHGTDTMDLSAAYID
CP003920.1_[2027875_2028355]_-      ILYKDSLEMDNHDRSLLAQTIAQRN----EKIIIVVHGTDTMDLSAEYVA
CP000361.1_[1593264_1593804]_-      IISKDSLEIVDEDRDILVETIKKLE----NENIIIVHGTDTIDITAKYLS
CP006615.1_[1549239_1549779]_-      IISKDSLEIVDEDRDILVETIKKLE----NENIIIVHGTDTIDITAKYLS
AP012047.1_[1512171_1512675]_-      IISKDSLEIIDEDRDILVETIKKLE----NENIIVVHGTDTIDITAKYLN
AP012048.1_[2108336_2108825]_-      VISKDSLEIDNDDRDYLVKIINES-----SENIIIVHGTDTIDITSKYID
CP001999.1_[1168204_1168696]_+      IVSKDSLYFTDEDRETILKEVQNST----SDKIIIIHGTDTVDLTSKFFD
CP003333.1_[452133_452628]_-_       LIHKDSLEMNEEDREELLKAIEASS----SHKILIIHGTDTMDVTASFLA
CP007201.1_[577476_577971]_-_       IIHKDSLEMSEEDRELIVQTIQASP----ARKVLIIHGTDTMDVTATFLA
CP001816.1_[424310_424805]_-_       ILHKDSLEMNDADRAHIAHTIQTST----SHKILIVHGTDTMDQTARFLA
AP009179.1_[734918_735413]_-_       IIGKDSLAMTNQDRLELLSVATQTE----HEKIIIIHGTDTMHVTAEYLA
CP001801.1_[2047872_2048406]_+      LIHKDSLDMTQSDRALICASIRNSLPAFNSAPVIIIHGTDTMHETALFLD
CP002930.1_[3753879_3754398]_+      LLSKDSLYMTDEDRALISATVKDQMQ--RGSPIVVLHGTDTMHISAEHCF
FQ312005.1_[1251955_1252498]_+      ILAKDSLNMTDYDRALVAKSLKVQLE--KGWPIIVLHGTDTMAKTAEYCF
CP002116.1_[274963_275503]_-_       VMSKDSLEMVDADRELILREVRAALP--VSDAIIIVHGTDTLSKTGECIY
CP002467.1_[871730_872243]_-_       LMNKDSLVMTPEDRELIATAAARSTS--RGIPVVVTHGTDTMVQTGLVLA
CP003379.1_[4474444_4474963]_+      LMNIDSLVMTNDHRKLIAERVLAQAT--EGTPVLVTHGTDTVVETGKVVA
CP000394.1_[466380_466920]_-_       PVLKDSLDFTDADRDAVADAVFGAESR--FGGILVTHGTDTVRETMRHLE
CP003181.1_[468418_468958]_-_       PVLKDSLDFTDADRDAVADAVFGAESR--FGGILVTHGTDTVRETMRHLE
CP003182.1_[471874_472414]_-_       PVLKDSLDFTDADRDAVADAVFGAESR--FGGILVTHGTDTVRETMRHLE
CP003183.1_[466403_466943]_-_       PVLKDSLDFTDADRDAVADAVFGAESR--FGGILVTHGTDTVRETMRHLE
AP011115.1_[2298178_2298610]_-      FVEGTDVTMGDGFRWVTILHPDHPE----LEVTLMRPGPPLDADMAEAIR
CP000431.1_[2563714_2564146]_-      FAEGTDVTMGDGFRWVTILHPDHPE----LEVTLMKPGPPLDADMAEAIR
                                         .  .                        ::  *.
```

```
CP006890.1_[255482_256010]_+_      --------------LTVKSDK-------------PVVVAGSMRPSTPQGR
FR775239.1_[132829_133354]_-_      AYPIRAI---LLAILVLGSAQNPHVL-RVRSGFCALSVSKLAAPITPTGI
CP009351.1_[884050_884524]_+_      -YMLQGL---RKPVILTGSQVPISFK-KTDAKKNVADALRFAC-EDVGGV
FP929062.1_[1670842_1671370]_+     -YLVQHS---SKPIVITGAQKPIDLD-VTDARTNLLDSLRFAASERAHGV
CP000407.1_[334649_335075]_-_      --TMAIP---EKPIVLTGAMRSSNEL-GSDGIYNYRTALRVATDDNPQIQ
CP003778.1_[4072337_4072823]_+     TIAD-------KTIVMTGALNPARFR-GSDAEFNIGCAVG-AVQSLPAGV
AE008923.1_[1096145_1096631]_-     TIAD-------KTIVMTGALNPARFR-GSDAEFNIGCAVG-AVQSLPAGV
CP004399.1_[1100060_1100546]_-     TIAD-------KTIVMTGALNPARFR-GSDAEFNIGCAVG-AVQSLPAGV
CP006857.1_[1096139_1096625]_-     TIAD-------KTIVMTGALNPARFR-GSDAEFNIGCAVG-AVQSLPAGV
CP008989.1_[1096143_1096629]_-     TIAD-------KTIVMTGALNPARFR-GSDAEFNIGCAVG-AVQSLPAGV
CP008992.1_[1096143_1096629]_-     TIAD-------KTIVMTGALNPARFR-GSDAEFNIGCAVG-AVQSLPAGV
CP008995.1_[1096145_1096631]_-     TIAD-------KTIVMTGALNPARFR-GSDAEFNIGCAVG-AVQSLPAGV
CP008998.1_[1096147_1096633]_-     TIAD-------KTIVMTGALNPARFR-GSDAEFNIGCAVG-AVQSLPAGV
CP009004.1_[1096155_1096641]_-     TIAD-------KTIVMTGALNPARFR-GSDAEFNIGCAVG-AVQSLPAGV
CP009001.1_[1096150_1096636]_-     TIAD-------KTIVMTGALNPARFR-GSDAEFNIGCAVG-AVQSLPAGV
CP009007.1_[1096136_1096622]_-     TIAD-------KTIVMTGALNPARFR-GSDAEFNIGCAVG-AVQSLPAGV
CP009010.1_[1096138_1096624]_-     TIAD-------KTIVMTGALNPARFR-GSDAEFNIGCAVG-AVQSLPAGV
CP009016.1_[1096154_1096640]_-     TIAD-------KTIVMTGALNPARFR-GSDAEFNIGCAVG-AVQSLPAGV
CP009013.1_[1096126_1096612]_-     TIAD-------KTIVMTGALNPARFR-GSDAEFNIGCAVG-AVQSLPAGV
CP009019.1_[1096155_1096641]_-     TIAD-------KTIVMTGALNPARFR-GSDAEFNIGCAVG-AVQSLPAGV
CP009022.1_[1096145_1096631]_-     TIAD-------KTIVMTGALNPARFR-GSDAEFNIGCAVG-AVQSLPAGV
CP009028.1_[1096145_1096631]_-     TIAD-------KTIVMTGALNPARFR-GSDAEFNIGCAVG-AVQSLPAGV
CP009025.1_[1096140_1096626]_-     TIAD-------KTIVMTGALNPARFR-GSDAEFNIGCAVG-AVQSLPAGV
CP009031.1_[4072159_4072645]_+     TIAD-------KTIVMTGALNPARFR-GSDAEFNIGCAVG-AVQSLPAGV
CP009034.1_[4072160_4072646]_+     TIAD-------KTIVMTGALNPARFR-GSDAEFNIGCAVG-AVQSLPAGV
CP009037.1_[4072154_4072640]_+     TIAD-------KTIVMTGALNPARFR-GSDAEFNIGCAVG-AVQSLPAGV
CP009040.1_[4072108_4072594]_+     TIAD-------KTIVMTGALNPARFR-GSDAEFNIGCAVG-AVQSLPAGV
AM039952.1_[1094639_1095125]_-     TIAD-------KTIVMTGALNPARFR-GSDAEFNIGCAVG-AVQSLPSGV
CP002914.1_[1035664_1036150]_-     TIAD-------KTIVMTGALNPARFR-GSDAEFNIGCAVG-AVQSLPSGV
CP003057.1_[982067_982553]_-_      TIAD-------KTIVMTGALNPARFR-GSDAEFNIGCAVG-AVQSLPAGV
CP007221.1_[4092673_4093159]_+     TIAD-------KTIVMTGALNPARFR-GSDAEFNIGCAVG-AVQSLPAGV
FO681494.1_[1080795_1081281]_-     TIAD-------KTIVMTGALNPARFR-GSDAEFNIGCAVG-AVQSLPAGV
AP008229.1_[3882331_3882817]_+     TIAG-------KTIVMTGALNPARFR-GSDAEFNIGCAVG-AVQSLPAGV
CP007166.1_[1135333_1135819]_-     TIAG-------KTIVMTGALNPARFR-GSDAEFNIGCAVG-AVQSLPAGV
CP011256.1_[5126005_5126491]_-     SIAD-------KTIVMTGALNPARFR-GSDAEFNIGCAVG-AVQSLPAGV
AE008922.1_[1016302_1016788]_-     SIAD-------KTIVMTGALNPARFR-GSDAEFNIGCAVG-AVQTLPAGV
CP000050.1_[4023752_4024238]_+     SIAD-------KTIVMTGALNPARFR-GSDAEFNIGCAVG-AVQTLPAGV
CP010409.1_[2013868_2014354]_+     AIEG-------KTIVMTGALNPARFR-GSDAEFNIGCAVG-AVQSLPAGV
FP565176.1_[530509_530995]_-_      TLQN-------KTIVMTGALNPARFR-GSDAEFNIGCAVG-AVQSLPPGV
CP002986.1_[819984_820479]_-_      TIPD-------KTIVMTGALSPARFR-GSDAEFNIGCAIG-AVQSLPSGV
HE798556.1_[847871_848366]_-_      TIPD-------KTIVMTGALSPARFR-GSDAEFNIGCAIG-AVQSLPSGV
AM743169.1_[902805_903291]_-_      TIPD-------KTIVMTGALSPARFR-GSDAEFNIGCAIG-AVQSLPSGV
CP001111.1_[830691_831186]_-_      TIPE-------KTIVMTGALSPARFR-GSDAEFNIGCAIG-AVQSLPSGV
CP002446.1_[2592481_2592967]_+     SIPD-------KTIVMTGALSPARFR-GSDAEFNIGCAIG-AVQTLPAGV
CP011144.1_[894375_894861]_+_      SIAD-------KTIVMTGALSPARFR-GSDAEFNIGCAIG-AVQSLPHGV
AE009442.1_[474685_475171]_-_      SIQN-------KTIVITGALNPARFH-GSDAHFNIGCAIG-ALQSLSSGV
CP001011.1_[474538_475024]_-_      SIQN-------KTIVITGALNPARFH-GSDAHFNIGCAIG-ALQSLSSGV
CP002165.1_[1594542_1595028]_-     SIQN-------KTIVITGALNPARFH-GSDAHFNIGCAIG-ALQSLSSGV
AE003849.1_[1047304_1047790]_-     SIQN-------KTIVITGALNPARFH-GSDAHFNIGCAIG-ALQSLSSGV
CP000941.1_[485900_486386]_-_      SIQN-------KTIVITGALNPARFH-GSDAHFNIGCAIG-ALQSLSSGV
CP006740.1_[275355_275841]_-_      SIQN-------KTIVITGALNPARFH-GSDAHFNIGCAIG-ALQSLSSGV
CP003093.2_[2619491_2619977]_+     SLQD-------RTIVLTGALNPAGFR-GSDAEFNIGCAVG-AVQTAPNGV
CP003470.1_[1046951_1047443]_-     SIKG-------KVIVLTGALNPARFQ-GSDAVFNIGCAVA-AVQTLPDGV
CP008884.1_[4334510_4335002]_+     SIKG-------KVIVLTGALNPARFQ-GSDAVFNIGCAVA-AVQTLPDGV
CP007444.1_[3166036_3166528]_+     QIKG-------KVIVLTGALNPARFQ-GSDAVFNIGCAVA-AVQTLPDGV
CP003350.1_[866528_867014]_-_      AVTD-------KTIVLTGALNPARFM-GSDAVFNIGCAVS-AVQLLPPGV
AP010904.1_[661932_662448]_+_      DIPG-------KTIVLTGAMSPARFA-ASDAPFNVGCAVG-AVWSSPPGV
CP003746.2_[1122651_1123134]_+     GIKG-------KTIVLMGAMYPAEFR-DSDAVFNLGAAIM-AAQTLPEGV
CP003985.1_[747499_748000]_-_      GISD-------KTIVMTGSMYPAQFR-DSDAVFNIGCAVT-AAQTLGPGV
CP001807.1_[750839_751322]_+_      GIPG-------KTIVLVGSLSPARFK-SSDAEFNIGFALA-AVQTLPPGV
CP003029.1_[2540593_2541118]_-     GIPG-------KTIVLVGSLSPARFK-SSDAEFNIGFALA-AVQTLPPGV
CP002361.1_[27336_27843]_+_        GVPD-------KTIVLVGAMQPARFL-GSDAEFNIGYAVA-AVQLLPPGV
CP000356.1_[475998_476481]_+_      GIEG-------KTIVLTGAMQPASVR-ASDAEFNVGFALA-AAQTLPPGV
CP009122.1_[1397954_1398446]_+     GIAS-------KTIVITGAMQPASVR-ASDAEFNVGFALA-AVQTLPPGV
CP000514.1_[3980723_3981203]_-     SVAD-------RTIVLTGAMQPARMR-RTDAIFNIGFAWA-AVSLLPAGV
FO203363.1_[3607326_3607806]_-     SVAD-------RTIVLTGAMQPARMR-RTDAIFNIGFAWA-AVSLLPAGV
CP001978.1_[3522100_3522580]_-     GLKD-------KTIVLTGAMQPARMR-RTDAVFNVGFAWA-AVQLLPPGV
CP007151.1_[1684828_1685308]_+     KLEG-------KTIVLIGAMQPARMR-RTDALFNIGFAWS-AVQLLAPGV
AP010803.1_[2584265_2584769]_-     AIAG-------KTVVLVGALAPARFG-ESDASFNLGMAFA-TAQLAAPGV
CP002798.1_[1485579_1486083]_-     AIPG-------KTVVLVGALAPARFG-ESDASFNLGMAFA-TVQVAKPGV
AP012222.1_[2800176_2800680]_-     PLAQD------RAVVLVGALAPARFA-ESDAPFNLGMAFA-TAQIAPPGV
CP009291.1_[3253827_3254319]_-     HLTD-------KTVVLAGALAPARFS-ESDAAFNLGMAFA-CCQAASPGV
FR856862.1_[1855341_1855833]_-     HLTD-------KTVVLAGALAPARFS-ESDAAFNLGMAFA-CCQAASPGV
CP000248.1_[599766_600258]_+_      PIEG-------KTIVLTGALSPARFA-ETDAPFNLGMAFA-TAQVAAPGI
CP001087.1_[2132169_2132652]_-     AVKG-------KTIVLTGAMEPAGFK-SSDATFNLGFAIA-AAQTLSHGV
FO203503.1_[3427664_3428150]_+     DIKN-------KVIVLTGAMEPAKFK-SSDAIFNIGFAVA-AVQTLPHGV
CP000230.1_[4281964_4282483]_+     GLPELA----GKTVVLSGAMVPGRVG-GSDAAFNIGFACA-AALMLAPGV
CP003046.1_[4281964_4282483]_+     GLPELA----GKTVVLSGAMVPGRVG-GSDAAFNIGFACA-AALMLAPGV
CP001131.1_[1967759_1968263]_+     AAG------LGKTVVLTGAMIPYAFG-SSDGLFNLGSALS-FVQVLPPGV
CP001359.1_[2029760_2030264]_+     AAG------LGKTVVLTGAMIPYAFG-SSDGLFNLGSALS-FVQVLPPGV
CP000251.1_[2419660_2420164]_-     AAG------LGKTVVLTGAMIPYAFG-SSDGLFNLGSALS-FVQVLPPGV
CP000769.1_[4112838_4113333]_+     AAE------TGKTVVLTGAMVPYAFG-SSDGLFNLGSALS-FAQVLPPGV
AP009153.1_[2726509_2727019]_-     ATI------TGKTIVLTGAMVPYAFG-SSDGLFNLGSALS-FAQTLPEGV
CP002691.1_[6510574_6511072]_-     NAN-----IENKTIVLTGAMIPYAFGTSSDGFFNMGSALA-FVQMLPPGI
CP002831.1_[2952159_2952681]_-     KAG---K-IEGKTIVLLGAMIPYTFGMSSDGFFNLGSAIA-FVQTLPPGV
CP003178.1_[7878322_7878829]_-     KK------VTGKTVVLTGAMIPIKFG-SSDGLFNLGSALA-FVQSLPPGV
CP007035.1_[3690700_3691195]_-     ER------IKNKTIVLTGAMIPIKFG-SSDGFFNLGSAIA-FAQSLPHGV
CP000473.1_[8450546_8451041]_+     PAL------PEKTIVLTGAMVPYTFG-SSDGLFNLGTAMA-FVQTLEPGV
CP001734.1_[541069_541603]_+_      GNA------LEKTIALVGAMIPYTFG-RSDALFNLGCAFS-TVQVMGHGV
CP003221.1_[3710374_3710884]_+     PAV------TGKAVALVGAMIPYSFV-HSDAGFNLGCAVT-AVQCLPPGV
CP002514.1_[2380426_2380936]_+     AQV------TDKTVVFVGAMIPYAFG-GSDALFNLGFALA-SARTLPPGV
CP000269.1_[4096942_4097428]_+     ----AA--QLPKTIVLTGAMIPYEVK-NSDALFNFGFACG-IAQALPHGV
CU207211.1_[3414611_3415097]_+     ----AA--QLPKTIVLTGAMVPYEVK-NSDALFNFGFACG-IAQALPHGV
CP002745.1_[5163657_5164143]_+     ----AA--ALEKTIVVTGAMIPYAIA-NSDALFNFGFACG-VAQALPHGV
CP009962.1_[5575893_5576379]_+     ----AA--ALEKTIVVTGAMIPYAIV-NSDALFNFGFACG-VAQALPHGV
HG322949.1_[136787_137273]_-_      ----GA--GLTAAVVLTGAMIPYEID-NSDALFNLGFACG-VAQVLPPGV
CP002039.1_[4937972_4938458]_+     ----QA--LTGKTVVLTGAMIPYEIA-NSDALFNFGFACG-VAQVLPAGV
CP002868.1_[1048763_1049252]_+     ----PA--SLNKTIVLTGAMIPYSVA-GSDSLFNLGCAFT-AVQLLSPGV
CP003282.1_[779163_779724]_+_      ----QA--GLDAAIVLTGAMIPYSIS-NSESLFNLGSAIA-AAGILPPGV
CP002541.1_[562683_563187]_-_      ETLGKE--NT-KTVVLTGAMIPYSLE-GSDAIFNLGTAIA-SVQLLPSGV
CP003155.1_[1580411_1580912]_+     EAMGKE--NT-HVIVFTGAMIPYSLE-NSDAVFNLGCAIT-AVQTLKAGV
CP002659.1_[299546_300053]_+_      RTLGPG--DQ-HVVVFTGAMIPYSLE-GSDAAFNLGSAMT-AAQVLTPGV
CP006939.1_[1834369_1834888]_+     KRFQEE--GVSRTVVFTGAMVPYSVN-GSDALFNLGAAVS-AAQLLDEGI
AP009247.1_[226235_226721]_+_      K-----E-INNKTIVLFGSMIPYSIN-CSDALFNLGVALS-AVQIKEYGV
CP001100.1_[295450_295954]_+_      Q-----S-LKGKTVVLFGAMIPYSFSTNTDALFNFGFALA-AVQLAPSGV
CP002776.1_[888646_889153]_-_      NRA--AN-LAQKTLILTGAMRPFKLG-QSDASFNLGAALM-AVQHLPADV
CP007030.1_[1079366_1079873]_+     DRL--AN-LPHKTLVLTGAMRPFQLG-QSDASFNLGAALM-AAQLLPAGV
CP000109.2_[1350751_1351261]_+     N-S--HQ-LTDKTIVLTGAMRPFQLN-QSDASFNLGAALM-ASQLASPGI
CP002959.1_[998274_998760]_-_      AAD------IAKTIVLTGAIVPYMAP-ESDATFNLGFALA-CAQTLAPGV
CP004393.1_[4207116_4207608]_-     GQV------TGKTVVLTGAMRPFSFG-ASDGPFNLGGAVI-AAQHLPEGV
CP001650.1_[1932398_1932884]_-     KLN------LKKTIVLVGSFILGSSA-DTDAPFNLGYAIS-SLQLLKPDV
CU207366.1_[1270492_1270984]_+     KEE------LKKTIVLVGSFILGSSA-NTDASFNLGYAIC-ALQFLKPDV
AP011540.1_[190666_191188]_+_      EHADLG----NKVVVLTGAMQPAVMA-RSDAGFNLGAAIA-ALNLLEPGV
CP010848.1_[381590_382076]_-_      AVSDR-------VIVLTGAMQPARMR-DSDASFNLGTAIA-AVQLLPPGV
AP009178.1_[1477696_1478179]_-     PYIH------NKCVVFTGAMVPYSID-PAEASANLALAISKVQYAYEDGV
CP002452.1_[1342864_1343380]_+     RHCP------DRQIVLAGAMIPYSID-PVEATANFASAIGWLLGNDAPGV
CP000153.1_[583322_583808]_+_      EVFD------DRKIVFVGSMRPFEID-NIEASLNLGMAIGFAKALKENGV
CP002205.1_[1564141_1564627]_-     EVFD------DRKIILTGAMKPFEID-YIEASLNLGMAIGYAKAVEMNGV
CP002355.1_[2175798_2176278]_-     MLAL------DKTVVFTGSMVPFSID-PIEATANLSMAIGYGQHA-VRGV
CP003920.1_[2027875_2028355]_-     SLSL------DKVVVFTGAMVPFSID-PVEATANLSMAIGYANNG-SAGV
CP000361.1_[1593264_1593804]_-     DRIE------NKKIVLTGAMVPMSIK-KVEATLNFAQAIGFLNASIQNGV
CP006615.1_[1549239_1549779]_-     DRIE------NKKIVLTGAMVPMSIK-KVEATLNFAQAIGFLNASIQNGV
AP012047.1_[1512171_1512675]_-     NRIE------NKKIVLTGAMVPMSIK-KVEATLNFAQAIGFLNAKISNGI
AP012048.1_[2108336_2108825]_-     ERVK------DKKIVLTGAMVPMSIK-KVEATLNFAQAIGFLNAPISNGV
CP001999.1_[1168204_1168696]_+     GKIE------GKVIVFTGAMVPMSID-EVEATMNFSQALGFLNAEVKPGI
CP003333.1_[452133_452628]_-_      LHVK------NKIITLTGAMVPFSID-TVEATANVMLALGDLHVRENNGV
CP007201.1_[577476_577971]_-_      LHVK------DKIISLTSAMVPFSID-TVEATANFMMALGDLHVREKNGV
CP001816.1_[424310_424805]_-_      LHVK------EKCVILTGAMVPFSID-TTEATANFMFALGALHVTEKKGI
AP009179.1_[734918_735413]_-_      EVEI------EKKIILTGAMVPYSID-PVEATANLTSALGYMQLLEEDGV
CP001801.1_[2047872_2048406]_+     EENL------NRVIVITGAMRPAEID-PVEASLHLGLTLGFAAANPPPGI
CP002930.1_[3753879_3754398]_+     KDIGT----PKVPVVFTGAMIPMGFD-DSDAAQNVTEALL-SAKLLQPGF
FQ312005.1_[1251955_1252498]_+     EELKN----ITSPIVFTGAMKPMGFD-DSDARQNVTEALL-ASKLLKPGV
CP002116.1_[274963_275503]_-_      QNIQD----STIPIILTGAMRPYEFR-DSDALQNITEALF-AAKVASPGI
CP002467.1_[871730_872243]_-_      DRLPDLR----TPVLLTGAMTPFGIE-GSDAIQNLTESLL-AASLVAPGV
CP003379.1_[4474444_4474963]_+     ETLAQHSAAATVPVVFTGAMTPFGIE-GSDALQNLTEALL-ATRMLPGGV
CP000394.1_[466380_466920]_-_      ARVAAGKAVLTRPVVFTGAMAPLHID-GSDAVQNLSAALT-ALRLLPPGL
CP003181.1_[468418_468958]_-_      ARVAAGKAVLTRPVVFTGAMAPLHID-GSDAVQNLSAALT-ALRLLPPGL
CP003182.1_[471874_472414]_-_      ARVAAGKAVLTRPVVFTGAMAPLHID-GSDAVQNLSAALT-ALRLLPPGL
CP003183.1_[466403_466943]_-_      ARVAAGKAVLTRPVVFTGAMAPLHID-GSDAVQNLSAALT-ALRLLPPGL
AP011115.1_[2298178_2298610]_-     RSLA-------SGTMGGFGLTTTDCHETYRDLVAKGVEFTQPPAERPYGI
CP000431.1_[2563714_2564146]_-     RSLA-------AGTMGGFGLTTTDCHETYKDLVAKGVEFTQPPAERPYGV
                                                          .
```

```
CP006890.1_[255482_256010]_+_       ETER-----------------------------------
FR775239.1_[132829_133354]_-_       GS-------------------------------------
CP009351.1_[884050_884524]_+_       FIVFDSRVIIGTRAVKM----------------------
FP929062.1_[1670842_1671370]_+      TIVFDGKVIAGTRGKRSAARATTPSPASTSRTSP-----
CP000407.1_[334649_335075]_-_       VF-------------------------------------
CP003778.1_[4072337_4072823]_+      YIAMNGRIWNPQKVRKNVAANRFESV-------------
AE008923.1_[1096145_1096631]_-      YIAMNGRIWNPQKVRKNVAANRFESV-------------
CP004399.1_[1100060_1100546]_-      YIAMNGRIWNPQKVRKNVAANRFESV-------------
CP006857.1_[1096139_1096625]_-      YIAMNGRIWNPQKVRKNVAANRFESV-------------
CP008989.1_[1096143_1096629]_-      YIAMNGRIWNPQKVRKNVAANRFESV-------------
CP008992.1_[1096143_1096629]_-      YIAMNGRIWNPQKVRKNVAANRFESV-------------
CP008995.1_[1096145_1096631]_-      YIAMNGRIWNPQKVRKNVAANRFESV-------------
CP008998.1_[1096147_1096633]_-      YIAMNGRIWNPQKVRKNVAANRFESV-------------
CP009004.1_[1096155_1096641]_-      YIAMNGRIWNPQKVRKNVAANRFESV-------------
CP009001.1_[1096150_1096636]_-      YIAMNGRIWNPQKVRKNVAANRFESV-------------
CP009007.1_[1096136_1096622]_-      YIAMNGRIWNPQKVRKNVAANRFESV-------------
CP009010.1_[1096138_1096624]_-      YIAMNGRIWNPQKVRKNVAANRFESV-------------
CP009016.1_[1096154_1096640]_-      YIAMNGRIWNPQKVRKNVAANRFESV-------------
CP009013.1_[1096126_1096612]_-      YIAMNGRIWNPQKVRKNVAANRFESV-------------
CP009019.1_[1096155_1096641]_-      YIAMNGRIWNPQKVRKNVAANRFESV-------------
CP009022.1_[1096145_1096631]_-      YIAMNGRIWNPQKVRKNVAANRFESV-------------
CP009028.1_[1096145_1096631]_-      YIAMNGRIWNPQKVRKNVAANRFESV-------------
CP009025.1_[1096140_1096626]_-      YIAMNGRIWNPQKVRKNVAANRFESV-------------
CP009031.1_[4072159_4072645]_+      YIAMNGRIWNPQKVRKNVAANRFESV-------------
CP009034.1_[4072160_4072646]_+      YIAMNGRIWNPQKVRKNVAANRFESV-------------
CP009037.1_[4072154_4072640]_+      YIAMNGRIWNPQKVRKNVAANRFESV-------------
CP009040.1_[4072108_4072594]_+      YIAMNGRIWNPQKVRKNVAANRFESV-------------
AM039952.1_[1094639_1095125]_-      YIAMNGRIWNPQKVRKNVAANRFESV-------------
CP002914.1_[1035664_1036150]_-      YIAMNGRIWNPQKVRKNVAANRFESV-------------
CP003057.1_[982067_982553]_-_       YIAMNGRIWNPEKVRKNVAANRFESV-------------
CP007221.1_[4092673_4093159]_+      YIAMNGRIWNPEKVRKNVAANRFESV-------------
FO681494.1_[1080795_1081281]_-      YIAMNGRIWNPEKVRKNVAANRFESV-------------
AP008229.1_[3882331_3882817]_+      YIAMNGRIWKPEKVRKNVAANRFESV-------------
CP007166.1_[1135333_1135819]_-      YIAMNGRIWKPEKVRKNVAANRFESV-------------
CP011256.1_[5126005_5126491]_-      YIAMNGRIWNPEKVRKNVAANRFESL-------------
AE008922.1_[1016302_1016788]_-      YIAMNGRIWNPETVRKNVAANRFEPV-------------
CP000050.1_[4023752_4024238]_+      YIAMNGRIWNPETVRKNVAANRFEPV-------------
CP010409.1_[2013868_2014354]_+      YIAMNGRIWDPQKVRKNVAANRFEAA-------------
FP565176.1_[530509_530995]_-_       YIAMNGQIWDPQKVRKNVAANRFEAI-------------
CP002986.1_[819984_820479]_-_       YIAMNGRIFDPQHVRKNVAANRFESV-------------
HE798556.1_[847871_848366]_-_       YIAMNGRIFDPQHVRKNVAANRFESV-------------
AM743169.1_[902805_903291]_-_       YIAMNGRIFDPQHVRKNVAANRFESV-------------
CP001111.1_[830691_831186]_-_       YIAMNGRIFDPQHVRKNVAANRFESV-------------
CP002446.1_[2592481_2592967]_+      YIAMNGLIWDPARVRKNVAANRFEPA-------------
CP011144.1_[894375_894861]_+_       YIAMNGLVWDPAKVRKNVAANRFEAV-------------
AE009442.1_[474685_475171]_-_       YIAMNGRIWHPDKVRKNIIKNCFETI-------------
CP001011.1_[474538_475024]_-_       YIAMNGRIWHPDKVRKNIIKNCFETI-------------
CP002165.1_[1594542_1595028]_-      YIAMNGRIWHPDKVRKNIIKNCFETI-------------
AE003849.1_[1047304_1047790]_-      YIAMNGRIWHPNKVRKNIIKNCFETI-------------
CP000941.1_[485900_486386]_-_       YIAMNGRIWHPDKVRKNIIKSCFETI-------------
CP006740.1_[275355_275841]_-_       YIAMNGRIWHPDKVRKNIIKNCFETI-------------
CP003093.2_[2619491_2619977]_+      WIAMNGRIWDPRKVRKNVAANRFEAA-------------
CP003470.1_[1046951_1047443]_-      YIAMNGRVWDPAKVRKNRDANRFEEVAG-----------
CP008884.1_[4334510_4335002]_+      YITMNGRVWDPAKVRKNRDANRFEESNG-----------
CP007444.1_[3166036_3166528]_+      YITMNGRVWDPLKVRKNREANRFEESAG-----------
CP003350.1_[866528_867014]_-_       HIAMNGRIWDPLSVRKNREANRFEEA-------------
AP010904.1_[661932_662448]_+_       YIAMSGRVFPAGAVRKNRKRGRFEDAAPAGGEG------
CP003746.2_[1122651_1123134]_+      YIAMNGRVFNPAKCVKNVAENRFEEA-------------
CP003985.1_[747499_748000]_-_       YIAMNGCIFMPDHARKNVEKNRFEEQTDNKAG-------
CP001807.1_[750839_751322]_+_       YIAMNGRIFHPDRVRKNREANRFEPI-------------
CP003029.1_[2540593_2541118]_-      YIAMNGRIFHPDRVRKNREANRFEPI-------------
CP002361.1_[27336_27843]_+_         YIAMNGQVFGAERVRKNRERLRFEAAP------------
CP000356.1_[475998_476481]_+_       YVAMNGMIFDPQRTVKDRAGGRFVQEG------------
CP009122.1_[1397954_1398446]_+      YVAMNGMIFDPEKTVKDRAGSRFVQEG------------
CP000514.1_[3980723_3981203]_-      YIAMNGEVFEAGSVRKNLAAQRFERA-------------
FO203363.1_[3607326_3607806]_-      YIAMNGEVFEAGSVRKNLAAQRFERA-------------
CP001978.1_[3522100_3522580]_-      YIAMNGEVFEAGAVRKNLKAQRFERT-------------
CP007151.1_[1684828_1685308]_+      YIAMNGEVFEAGSVRKNLKAQRFERT-------------
AP010803.1_[2584265_2584769]_-      YITMSGSVFRADKVMKDREKGAFVPIDE-----------
CP002798.1_[1485579_1486083]_-      YITMSGSVFRADKVMKDRVKGAFVPVGN-----------
AP012222.1_[2800176_2800680]_-      YITMNGTVFRAAEVVKDRAQGAFVAMP------------
CP009291.1_[3253827_3254319]_-      WITMNGSVFDGLKVRKDREAGTFVEV-------------
FR856862.1_[1855341_1855833]_-      WITMNGSVFDGLKVRKDREAGTFVEV-------------
CP000248.1_[599766_600258]_+_       WIAMSGQVFDGLKVRKDRAAGKFVAV-------------
CP001087.1_[2132169_2132652]_-      YIAISGRIFDPARVRKNRELKQFEEK-------------
FO203503.1_[3427664_3428150]_+      YLAISGKIFTPDNVMKNRILKQFEEKM------------
CP000230.1_[4281964_4282483]_+      YIAMHGKVFDPAKTRKNRGLGRFEPIDDQE---------
CP003046.1_[4281964_4282483]_+      YIAMHGKVFDPAKTRKNRGLGRFEPIDDQE---------
CP001131.1_[1967759_1968263]_+      YVAMNGRVFPGDKVRKNRQTGTFEDLA------------
CP001359.1_[2029760_2030264]_+      YVAMNGRVFPGDKVRKNRQTGTFEDLA------------
CP000251.1_[2419660_2420164]_-      YVAMNGRVFAGDKVRKNRQTGIFEDLA------------
CP000769.1_[4112838_4113333]_+      YVAMNGRVFPADRVRKNRESGIFEET-------------
AP009153.1_[2726509_2727019]_-      YIAMNGRYFAAGHVMKNREVGVFEESPNPPTSQD-----
CP002691.1_[6510574_6511072]_-      YVVMNGRYFNWDNVRKNRKTGNFETIHPE----------
CP002831.1_[2952159_2952681]_-      YIAMHGEYFNWDNVRKNRSTGYFETTKDKKN--------
CP003178.1_[7878322_7878829]_-      YVAMNGRFFNWDNVRKNRQTGIFEELPVPTAAK------
CP007035.1_[3690700_3691195]_-      YVAMNGQYFKASNVRKNRQTGVFEALQKI----------
CP000473.1_[8450546_8451041]_+      YVAMNGRCFPWHSVRKNKKEGVFELRRG-----------
CP001734.1_[541069_541603]_+_       YITMNGRIFYWDNVRKNTSIGDFELLEDPGTHDKER---
CP003221.1_[3710374_3710884]_+      YVTMNGRIFTWDNVRKNREIGEFGKLKE-----------
CP002514.1_[2380426_2380936]_+      YIAMNGTVFDWRNVRKNRERGRFEPLHP-----------
CP000269.1_[4096942_4097428]_+      YIAMNAQIFTWNNVRKNRSAGVFEKI-------------
CU207211.1_[3414611_3415097]_+      YIAMNAQIFTWNDVKKNRAEGVFEKR-------------
CP002745.1_[5163657_5164143]_+      YVAMNGKIFAWDKVEKNRVAGVFEPL-------------
CP009962.1_[5575893_5576379]_+      YVAMNGKIFAWDKVEKNRTAGVFEPL-------------
HG322949.1_[136787_137273]_-_       YVAMNGQIFAWDNVQKNRAAGVFQPL-------------
CP002039.1_[4937972_4938458]_+      YVAMNGRIFAWNKVAKNRSAGVFEPL-------------
CP002868.1_[1048763_1049252]_+      YITMNGRVFPWDKVRKNYEKGVFEGL-------------
CP003282.1_[779163_779724]_+_       YAAMNGRIFAGSEVRKDPRHGIFLGHKPTI---------
CP002541.1_[562683_563187]_-_       YLTMSGRIFSWDNCRKNKEKGIFETLT------------
CP003155.1_[1580411_1580912]_+      YICMSGRIFPWDNCIKNKDKGIFEYL-------------
CP002659.1_[299546_300053]_+_       YICMSGRVFTWDDCVKNKTKGIFETLT------------
CP006939.1_[1834369_1834888]_+      YICMNGRVFQAGKVMKDRGKGIFTDAETDG---------
AP009247.1_[226235_226721]_+_       YITMNGQVFDFDKVEKNKEIGIFTLL-------------
CP001100.1_[295450_295954]_+_       FIAMNGKLFNWDTVTKNKQKGVFQTVENA----------
CP002776.1_[888646_889153]_-_       YIVMNGQVFSAHQVQKDRSRGVFLANTEH----------
CP007030.1_[1079366_1079873]_+      YLVMNGQVFLARQVQKDRARGVFVPKRSA----------
CP000109.2_[1350751_1351261]_+      YIAMNGELFEASQVSKNRQHGIFELHT------------
CP002959.1_[998274_998760]_-_       YIAMNGKIFAWNNVRKNQQLKVFEPLR------------
CP004393.1_[4207116_4207608]_-      WGVMNGRVFAAEALDKNTGTGRFDT--------------
CP001650.1_[1932398_1932884]_-      YIAMNGQVLLWNNVSKNLETNKFE-RNE-----------
CU207366.1_[1270492_1270984]_+      YVVMNGRIFHWSNVTKNLESNKFEPKNE-----------
AP011540.1_[190666_191188]_+_       YISMSGRIFPAESVRKDRSRGIFEG--------------
CP010848.1_[381590_382076]_-_       YLAMSGRVLPAGAVVKNRDIGIFLPR-------------
AP009178.1_[1477696_1478179]_-      FIAMHGIVEQYDRVYKDRARGVFCRK-------------
CP002452.1_[1342864_1343380]_+      FIAMHGLVLPYDRIVKDREKGIFVHKA------------
CP000153.1_[583322_583808]_+_       YICMSGHVEPWQKIYKNKKFGKFEVVL------------
CP002205.1_[1564141_1564627]_-      YICMSGLVEPWKNIAKNRNLGKFEIVR------------
CP002355.1_[2175798_2176278]_-      HIVMQGVMNSFKQVKKNKSAGKFEYV-------------
CP003920.1_[2027875_2028355]_-      HIVMQGVMGNFVHVKKNKSAGKFEYV-------------
CP000361.1_[1593264_1593804]_-      YISMHGCVTNYAKLLKNRQLGQFLIEE------------
CP006615.1_[1549239_1549779]_-      YISMHGCVTNYAKLLKNRQLGQFLIEE------------
AP012047.1_[1512171_1512675]_-      YISMHGAVKSYDKLLKNRILGQFLIEE------------
AP012048.1_[2108336_2108825]_-      YISMHGCVTNYAKLLKNRQLGQFLIS-------------
CP001999.1_[1168204_1168696]_+      YISMHGVVVEHSRLKKDRSVGQFLIN-------------
CP003333.1_[452133_452628]_-_       YIAMHGRVAAHTQLYKNRTKGVFEPS-------------
CP007201.1_[577476_577971]_-_       YLAMHGAIASHGSIYKNRQKGIFELC-------------
CP001816.1_[424310_424805]_-_       YIAMHGAIAEHSHLYKNREKGIFERC-------------
AP009179.1_[734918_735413]_-_       YTVMNGLFASYDKIVKDKSKGKFVRKK------------
CP001801.1_[2047872_2048406]_+      YIAMHGRVLPYAEYRKNRTLGVFQTT-------------
CP002930.1_[3753879_3754398]_+      YISFHNQVFNVPQVRKNREKGTFESF-------------
FQ312005.1_[1251955_1252498]_+      YISFHNRVFSVPGVRKNRDKKTFEAF-------------
CP002116.1_[274963_275503]_-_       YAVMHNRLLPFPGVVKDRTSLTFRTQEEIEKISTNETKA
CP002467.1_[871730_872243]_-_       YIVFHGEVFPIADVKKDTEQSRFVRVSQR----------
CP003379.1_[4474444_4474963]_+      YLAFHGQVFPIANVRKDRENSRFLRID------------
CP000394.1_[466380_466920]_-_       HLAFHDQVLSGTQFEKDRTIGTFRQL-------------
CP003181.1_[468418_468958]_-_       HLAFHDQVLSGTQFEKDRTIGTFRQL-------------
CP003182.1_[471874_472414]_-_       HLAFHDQVLSGTQFEKDRTIGTFRQL-------------
CP003183.1_[466403_466943]_-_       HLAFHDQVLSGTQFEKDRTIGTFRQL-------------
AP011115.1_[2298178_2298610]_-      EAVMRDNSGNWLVLVEKREYTGEDFAH------------
CP000431.1_[2563714_2564146]_-      EAVMRDNSGNWLVLVEKREFTGEDFPH------------
```

Sequences were extracted from the European Nucleotide Archive, ENA (https://www.ebi.ac.uk/ena/browser/home), using nucleotide sequence of RrA as the probe. For each sequence, the first string (i.e. CP000230.1_[4281964_4282483]) represents the accession code for a particular entry and the extent of nucleotides encoding scASNase in that genome (within square brackets). For example, the accession code CP000230.1 refers to *Rhodospirillum rubrum* (RrA). Lines representing the RrA sequence are highlighted in yellow (one per page). Highly conserved lysine residue K19, unique to RrA but not to other Class 1 L-asparaginases, is shown in red.
